# Autoregressive Higher-Order Hidden Markov Models: Exploiting Local Chromosomal Dependencies in the Analysis of Tumor Expression Profiles

**DOI:** 10.1371/journal.pone.0100295

**Published:** 2014-06-23

**Authors:** Michael Seifert, Khalil Abou-El-Ardat, Betty Friedrich, Barbara Klink, Andreas Deutsch

**Affiliations:** 1 Center for Information Services and High Performance Computing, Dresden University of Technology, Dresden, Germany; 2 Institute for Clinical Genetics, Faculty of Medicine Carl Gustav Carus, Dresden University of Technology, Dresden, Germany; University of Pécs Medical School, Hungary

## Abstract

Changes in gene expression programs play a central role in cancer. Chromosomal aberrations such as deletions, duplications and translocations of DNA segments can lead to highly significant positive correlations of gene expression levels of neighboring genes. This should be utilized to improve the analysis of tumor expression profiles. Here, we develop a novel model class of autoregressive higher-order Hidden Markov Models (HMMs) that carefully exploit local data-dependent chromosomal dependencies to improve the identification of differentially expressed genes in tumor. Autoregressive higher-order HMMs overcome generally existing limitations of standard first-order HMMs in the modeling of dependencies between genes in close chromosomal proximity by the simultaneous usage of higher-order state-transitions and autoregressive emissions as novel model features. We apply autoregressive higher-order HMMs to the analysis of breast cancer and glioma gene expression data and perform in-depth model evaluation studies. We find that autoregressive higher-order HMMs clearly improve the identification of overexpressed genes with underlying gene copy number duplications in breast cancer in comparison to mixture models, standard first- and higher-order HMMs, and other related methods. The performance benefit is attributed to the simultaneous usage of higher-order state-transitions in combination with autoregressive emissions. This benefit could not be reached by using each of these two features independently. We also find that autoregressive higher-order HMMs are better able to identify differentially expressed genes in tumors independent of the underlying gene copy number status in comparison to the majority of related methods. This is further supported by the identification of well-known and of previously unreported hotspots of differential expression in glioblastomas demonstrating the efficacy of autoregressive higher-order HMMs for the analysis of individual tumor expression profiles. Moreover, we reveal interesting novel details of systematic alterations of gene expression levels in known cancer signaling pathways distinguishing oligodendrogliomas, astrocytomas and glioblastomas. An implementation is available under www.jstacs.de/index.php/ARHMM.

## Introduction

Copy number changes of genes are frequently found in different types of cancer [1]. Mutations such as duplications of oncogenes and deletions of tumor suppressor genes contribute together with single nucleotide polymorphisms, epigenetic alterations and other types of mutations to changes in gene expression programs triggering the development of cancer [2]. Broad and focal duplications and deletions of chromosomal regions are known to directly influence expression levels of underlying genes. Genes with increased copy numbers tend to show increased expression, whereas genes with reduced copy numbers tend to show reduced expression in tumors compared to healthy tissue (e.g. [3]–[6]). This coupling of gene copy numbers and gene expression levels leads to local chromosomal dependencies between gene expression levels providing the opportunity to develop improved methods for the analysis of individual tumor expression profiles.

Over the last years, several approaches have been developed for the analysis of tumor expression profiles in the context of chromosomal locations of genes. Methods like CGMA (comparative genomic microarray analysis) [7], MACAT (MicroArray Chromosome Analysis Tool) [8] or LAP (Locally Adaptive statistical Procedure) [9] require replicated measurements of tumor and normal reference samples for the identification of differentially expressed genes. Such methods cannot be applied to the analysis of individual tumor expression profiles in large screenings for which repeated profiling of the same sample is typically not done to reduce costs and to increase the number of screened tumors. Usually, log-fold change thresholds are used to determine differentially expressed genes in individual tumor expression profiles measured in such screenings. Alternatively, closely related methods for the analysis of comparative genomic hybridization data (e.g. reviewed and compared in [10] and [11]) can be applied to individual tumor expression profiles. For example, ChARM (Chromosomal Aberration Region Miner) [12] has also been demonstrated to identify differentially expressed chromosomal regions in individual tumor expression profiles. However, we have recently shown that both strategies only reach suboptimal performances that can be improved substantially by Hidden Markov Models (HMMs) utilizing prior knowledge on the distribution of gene expression measurements and chromosomal proximities of genes [13].

Generally, HMMs provide a sound mathematical grounding for the analysis of biological sequences [14], [15]. However, a current limitation of almost all existing HMM -based approaches is their limited potential to model local dependencies between measurements due to two restrictive assumptions. First of all, the commonly used standard first-order state-transition process only enables the modeling of local dependencies between directly adjacent hidden states. Secondly, it is commonly assumed that measurements only depend on the underlying state and do not directly influence each other. Nevertheless, HMMs with such restrictive assumptions have frequently been found to reach good results in a broad range of applications [14], [16], [17], but it has on the other hand also been demonstrated that the integration of a higher-order state-transition process can improve the model performance substantially in speech recognition (e.g. [18]), handwriting recognition (e.g. [19]), financial time-series analysis (e.g. [20]), image segmentation (e.g. [21]), robotics (e.g. [22]) and computational biology (e.g. [11], [23], [24]).

However, this only addresses the first limitation by overcoming the restrictive first-order state-transition process. Independent from that, extensions of the standard emission process have been initially realized in speech recognition by the integration of autoregressive emission distributions enabling the modeling of state-specific direct local dependencies within a range of successive measurements [25]. This concept has also been successfully applied in other domains including the analysis of financial time series [26], electrophysiological signals [27], evaluation of meteorological data [28], modeling of influenza and dengue fever epidemics [29], [30], speech synthesis [31] and for studying the locomotive behavior of flies [32]. However, there have been no general efforts to combine both concepts by realizing an HMM with higher-order state-transitions and autoregressive emissions.

**Figure 1:**
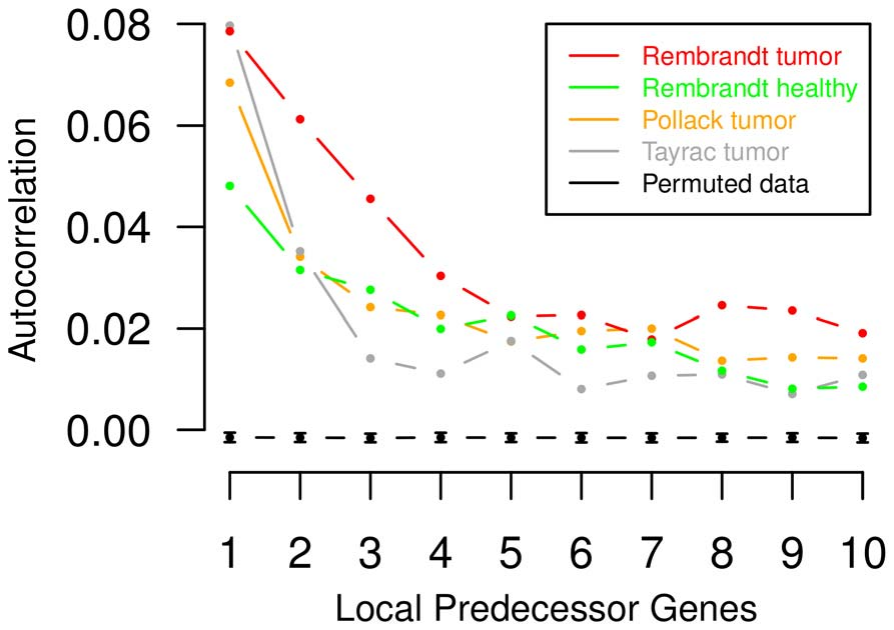
Local chromosomal dependencies of gene expression levels in different types of cancer. Spatial correlations of expression levels of genes in increasing chromosomal order up to ten were quantified by an average autocorrelation function that considers each chromosome-specific expression profile in each individual tumor sample. The autocorrelation function quantifies the similarity of gene expression levels of neighboring genes on a chromosome in a fixed distance. Corresponding average autocorrelation functions are shown for three types of cancer (i) different types of gliomas (red) [33], (ii) breast cancer expression profiles (orange) [3] and (iii) glioblastoma expression profiles (grey) [4]. Additionally, the green curve represents the average autocorrelation function of normal brain reference gene expression profiles taken from [33]. Due to chromosomal aberrations in gliomas, expression levels of genes in close chromosomal proximity tend to show greater similarity in gliomas (red) than in corresponding normal brain tissues (green). Moreover, the black curve represents mean values and standard deviations of the average autocorrelation function for randomly permuted glioma gene expression profiles from [33] across 100 repeats. The observation of significant local chromosomal dependencies in tumor expression profiles compared to permuted expression profiles motivates the development of autoregressive higher-order HMMs for the analysis of tumor expression profiles.

To overcome this, we develop a novel model class of autoregressive higher-order HMMs enabling an improved modeling of local dependencies between successive measurements. Autoregressive higher-order HMMs simultaneously utilize higher-order state-transitions in combination with autoregressive emissions as novel model features. Globally, this model class has very general modeling capabilities including mixture models, standard first-order HMMs and higher-order HMMs as special cases. We motivate the development of autoregressive higher-order HMMs by considering the analysis of individual tumor expression profiles in which local dependencies of gene expression levels are frequently caused by deletions and duplications of underlying chromosomal regions. The existence of such local chromosomal dependencies between expression levels of genes in close chromosomal proximity is clearly shown for three different types of cancer in Figure 1. Additionally, based on initial findings on the importance of integrating prior knowledge on the distribution of differentially expressed genes into the training of HMMs [13], we here also specifically design an efficient Bayesian Baum-Welch training for autoregressive higher-order HMMs.

We apply our autoregressive higher-order HMMs to the analysis of publicly available breast cancer and glioma gene expression data. We first systematically evaluate the performance of autoregressive HMMs to predict overexpressed genes in breast cancer with known underlying increased gene copy numbers. Then, we analyze to which extent these results are transferable to other similar breast cancer expression profiles. We further complement this by a sensitivity analysis evaluating the robustness of autoregressive HMM predictions. Next, we perform an in-depth comparison study to related existing methods on breast cancer gene expression profiles, where we first investigate general characteristics of predicted differentially expressed genes followed by the identification of overexpressed genes with known increased copy numbers. Finally, we consider different types of gliomas and demonstrate that autoregressive HMMs are useful tools to reveal systematic differences in expression levels of genes in known cancer signaling pathways.

## Materials and Methods

The materials part provides a brief overview of the considered data sets. The methods part gives a detailed description of the model class of autoregressive higher-order HMMs.

### Materials

This section shortly introduces the considered breast cancer and glioma data sets.

#### Breast Cancer Data

A breast cancer data set by *Pollack et al*. [3] is used to compare different bioinformatics methods for their potential to identify differentially expressed genes in breast cancer. This data set contains gene expression levels and corresponding gene copy numbers measured for 4 breast cancer cell lines and 37 tumors across 6,095 genes of the 23 human chromosomes. For each chromosome in each cell line and each tumor, we summarized the gene expression measurements in a chromosome-specific tumor expression profile 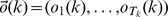 leading to 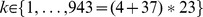 different profiles. Each profile represents the relative expression level of each gene 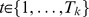 defined by the log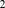-ratio 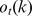 of its expression level in tumor divided by its corresponding expression level in the healthy reference sample. All log-ratios in a tumor expression profile are ordered from the p-arm to the q-arm of the underlying chromosome utilizing the chromosomal locations of the corresponding genes. A histogram of log-ratios of the breast cancer gene expression data set is shown in Figure S1 in Text S1. Local spatial dependencies between expression levels in the breast cancer gene expression profiles are shown in Figure 1.

#### Glioma Data

We created a glioma gene expression data set based on data from the Repository for Molecular Brain Neoplasia Data (Rembrandt, current release 1.5.9) [33] containing tumor samples of oligodendrogliomas (WHO grades II and III), astrocytomas (WHO grades II and III) and glioblastomas (glioblastoma multiforme: astrocytoma of WHO grade IV). We performed stringent quality controls of the downloaded gene expression arrays and removed all arrays with hybridization artifacts. We further did a standard Affymetrix microarray processing utilizing a customized design file from BrainArray (HGU133Plus2 version 15.0.0) in combination with GCRMA normalization [34]. The final data set contains tumor gene expression profiles of 89 different gliomas (45 glioblastomas, 33 astrocytomas, and 11 oligodendrogliomas) for which gene expression levels of 16,282 genes are quantified in terms of log-ratios with respect to an average normal brain reference computed based on data from Rembrandt. The individual tumor-specific chromosomal expression profiles were structured in analogy to the previously described breast cancer expression profiles. We use this data set to perform an in-depth comparison of gene expression changes affecting known cancer-relevant signaling pathways across different types of gliomas. Local chromosomal dependencies between gene expression levels in gliomas are shown in Figure 1.

### Methods

This section develops the basics of the model class of autoregressive higher-order HMMs in the context of the analysis of tumor expression profiles. This includes a detailed model description, a general characterization of the model class, the integration of prior knowledge into the training of the model, basics of the model training and the model initialization.

#### Autoregressive Higher-Order Hidden Markov Models

We develop a three-state HMM with state-specific autoregressive Gaussian emission densities for the identification of differentially expressed genes in tumors. The set of hidden states of the HMM is denoted by *S* :  = {‘ = ’,‘−’,‘+’}. Considering the usually observed distribution of log-ratios (e.g. Figure S1 in Text S1), genes with unchanged expression levels between tumor and normal tissue (log-ratios close to zero) are modeled by state ‘
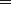
’. Underexpressed genes in tumor (log-ratios much less than zero) are modeled by state ‘
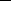
’, and overexpressed genes in tumor (log-ratios much greater than zero) are modeled by state ‘
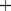
’. These states form the basis of the fully connected three-state architecture of the HMM illustrated in Figure S2 in Text S1.

We utilize a homogeneous higher-order Markov model (e.g. [35]) to model the state-transition process of the autoregressive HMM. This has recently been shown to improve the analyses of comparative genomics and DNA methylation data by a better modeling of spatial dependencies between closely adjacent chromosomal regions [11], [24]. The state-transition process of an HMM of order 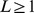 is specified by two components (i) an initial state distribution 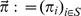 with initial state probability 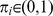 fulfilling the constraint 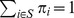 and (ii) a set of stochastic transition matrices 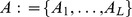. Each transition matrix 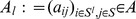 with 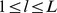 defines the transition probability 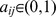 for each transition from the current state 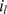 of a state-context 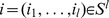 to a next state 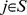. This means that for 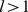 transitions from 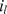 are depending on its 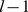 predecessors 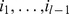. Each transition matrix 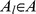 also fulfills the constraint 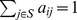 for each 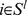. More formally, the state-transition process is used to model a hidden state sequence 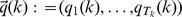 that underlies a specific tumor expression profile 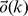. The individual state of a gene 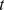 in a profile 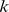 is denoted by 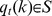. To model the state sequence 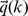, each transition matrix 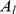 with 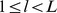 is used for the corresponding transition from the current state 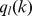 to the next state 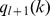 under consideration of the 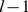 predecessor states 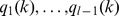. Finally, the transition matrix 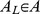 is used for each transition from 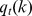 to 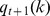 for all genes 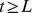 in dependency of the complete memory on 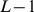 predecessor states 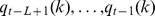.

In addition to the higher-order state-transition process, we also propose to model additional dependencies between emissions on the level of the observed tumor expression profile utilizing higher-order autoregressive Gaussian emission distributions. Similar to an initial work in speech recognition by *Ephraim et al*. [25], we adapt the concept of using an autoregressive emission process to the analysis of tumor expression profiles. For each hidden state 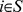, we assume that the expression level 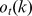 of a gene 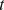 in a profile 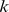 is modeled by a state-specific autoregressive Gaussian emission distribution of order 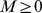 defined by
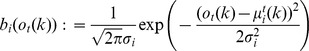
with respect to the state-specific standard deviation 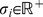 and the state-specific autoregressive mean
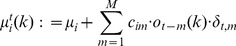
(1)for the expression level of gene 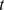 in profile 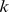. Here, 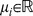 defines the basic state-specific mean and the coefficients 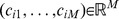 are used to model the impact of predecessor expression levels on the gene-specific mean. Additionally, at the start of an emission sequence, where the complete memory on 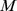 predecessor emissions does not exist, we have to truncate the modeling of dependencies by defining the function 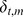 to be zero in cases where 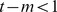 and otherwise one. The emission parameters are summarized in 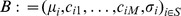. Additionally, all parameters of the autoregressive higher-order HMM are denoted by 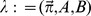.

The identification of differentially expressed genes is done by determining the probability with which each gene 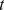 in a tumor expression profile 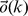 is assigned to a state 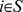 of the HMM. This state-posterior probability 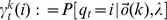 is computed using extended Forward-Backward algorithms adapted to higher-order HMMs [15]. The obtained state-posterior probabilities enable (i) the ranking of individual genes according to their potential of being differentially expressed and (ii) the determination of the most likely underlying state of each gene based on state-posterior decoding (e.g. [15], [36]). The autoregressive higher-order HMM has been implemented using Jstacs [37].

#### Existing Models Covered by Autoregressive Higher-Order Hidden Markov Models

**Figure 2:**
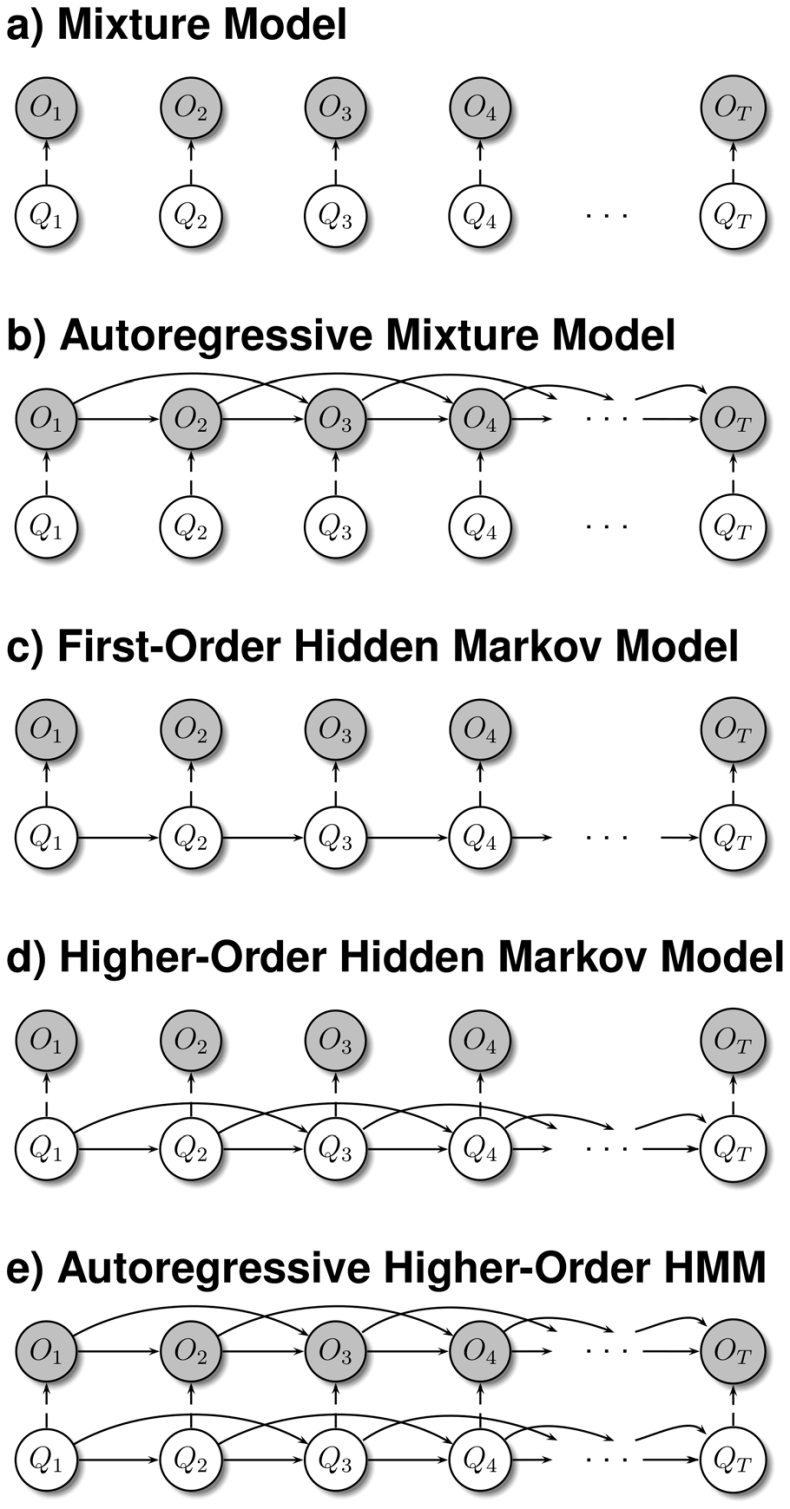
Selected state space representations of models included in the novel model class of autoregressive higher-order HMMs. State space representations of selected models included in the class of autoregressive higher-order HMMs. Hidden states are denoted by 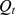 and emissions are denoted by 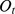 for 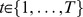. Arrows between nodes define modeled statistical dependencies. **a**) Standard mixture model (AR(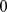)-HMM(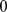)). **b**) Mixture model with second-order autoregressive emissions (AR(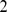)-HMM(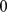)). **c**) Standard HMM with first-order state-transitions (AR(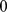)-HMM(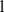)). **d**) Standard higher-order HMM with second-order state-transitions (AR(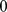)-HMM(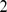)). **e**) Autoregressive higher-order HMM with second-order state-transitions and second-order autoregressive emissions (AR(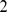)-HMM(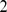)).

We introduce the notation AR(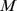)-HMM(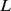) to specify an HMM with an autoregressive emission process of order 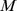 and a state-transition process of order 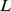. This model is part of the very general model class of autoregressive higher-order HMMs. This model class contains several special cases that have previously been studied in different domains also including applications in computational biology. Some selected underlying state space representations of specific models are shown in Figure 2 for increasing model complexities enabling a better modeling of dependencies between closely adjacent genes in a tumor expression profile.

Figure 2a represents the mixture model AR(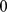)-HMM(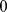) (e.g. [38]) that does not model any dependencies between genes. A second-order autoregressive mixture model AR(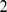)-HMM(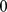) (e.g. [39]) is exemplarily shown in Figure 2b. This model only realizes dependencies between successive genes on the level of the measured gene expression levels. The standard first-order HMM AR(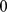)-HMM(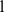), which is very popular in applied sciences (e.g. [15]–[17], [36]), is shown in Figure 2c. This model integrates dependencies between directly adjacent genes on a chromosome on the level of the hidden state-transitions of adjacent genes. A second-order HMM AR(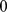)-HMM(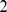) is exemplarily shown in Figure 2d. Such higher-order HMMs are still rarely used in practical applications, but they are known to be powerful extensions of first-order HMMs (e.g. [11], [18], [19], [24], [40]). Finally, a second-order HMM with second-order autoregressive emissions denoted by AR(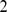)-HMM(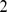) is shown in Figure 2e.

Generally, autoregressive higher-order HMMs implement a combination of higher-order state-transitions and autoregressive emissions to improve the modeling of local chromosomal dependencies between genes. The improved modeling of spatial dependencies is reached at the price of an increased number of model parameters and an increased computational complexity. For an HMM with 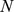 states, an autoregressive emission process of order 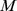 and a state-transition process of order 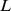, the processing of a tumor expression profile of length 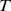 generally requires 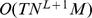 operations. The factor 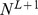 accounts for the state-transitions and the factor 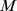 accounts for the emissions that have to be processed for each of the 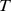 genes. Thus, in dependency of available training data, only small model orders should be considered in practical applications to avoid long training times and overfitting. For the analysis of microarray data, models with a state-transition process of order two up to four have shown the best performance in previous studies with higher-order HMMs [11], [24].

#### Integration of Prior Knowledge

The integration of prior knowledge into the training of HMMs has been shown to be a key feature for improving the identification of differentially expressed genes in tumors [13]. Thus, to achieve a problem-specific characterization of the autoregressive higher-order HMM, we define a prior distribution
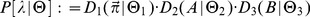
(2)over the space of the model parameters 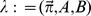 for given hyper-parameters 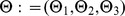. This prior distribution is specified by a product of three independent prior distributions enabling the integration of prior knowledge into the initial state distribution, the set of transition matrices and the emission parameters of the HMM. Following the usual choices of prior distributions for HMMs (e.g. [11], [13], [14], [24]), the prior 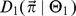 for the initial state distribution is defined by a Dirichlet distribution and the prior 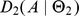 for the set of transition matrices is specified by products of Dirichlet distributions. Both priors represent conjugate priors enabling the analytical estimation of model parameters during the training. Details of both prior distributions are given in Appendix A in Text S1.

In the following, we summarize important characteristics of the emission prior 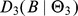 to enable the integration of prior knowledge into the training of the state-specific autoregressive Gaussian emission densities. To avoid that each gene 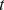 in a tumor expression profile 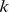 has its specific emission prior, we define the prior distribution for the state-specific autoregressive mean 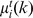 specified in Eqn. (1) using the following strategy. We only explicitly model prior knowledge for each basic state-specific mean 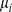 and assume constant priors for the corresponding coefficients 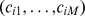. This leads to a reduction of the prior for 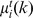 to a prior that is only depending on the corresponding 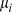. Based on that, the emission prior for an HMM with autoregressive Gaussian emissions can be defined by using a product of independent Gaussian-Inverted-Gamma distributions as used for non-autoregressive HMMs in [13]. Details of this emission prior in the context of autoregressive higher-order HMMs are provided in Appendix A in Text S1.

#### Bayesian Baum-Welch Training

The training of the autoregressive higher-order HMM is done by a Bayesian Baum-Welch algorithm enabling the integration of prior knowledge into the iterative parameter estimation process. This algorithm belongs to the class of Expectation-Maximization (EM) algorithms [41] and extends the typically used Baum-Welch training [36], [42] that does not utilize prior knowledge. In our context, the term ‘Bayesian’ specifies a Maximum-A-Posterior (MAP) estimate integrated into an EM algorithm. A specific version of a Bayesian Baum-Welch algorithm has been developed in [13] for first-order HMMs. This algorithm has been adapted to higher-order HMMs in [24]. Here, we further extend this algorithm to the requirements of autoregressive higher-order HMMs. The Bayesian Baum-Welch algorithm locally maximizes the log-posterior by a two-step procedure based on initially chosen model parameters. This is realized by iteratively determining new parameters
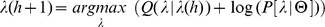
maximizing the log-posterior of the model 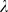 with respect to the current parameters of the model 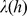 (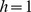 initial model). The log-posterior is specified by a combination of Baum's auxiliary function 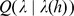 (Appendix B in Text S1; [13], [24], [36]) and the logarithm of the prior distribution 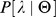 in Eqn. (2). This combination enables the iterative estimation of new model parameters under consideration of prior knowledge. The process of estimating new parameters 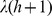 is iterated until the log-posterior increases less than a predefined threshold in comparison to the value obtained for the previous parameters 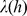. We stop the training if the increase of the log-posterior is less than 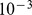 for two successive iterations.

Since we propose autoregressive Gaussian emissions as a novel model feature, we briefly summarize important steps of the estimation of the corresponding autoregressive means. The maximization step of the emission parameter estimation part of the log-posterior leads to a state-specific system of linear equations that enables to update the basic mean and the corresponding coefficients for each autoregressive mean. Standard solvers for systems of linear equations can be used to compute the solution of the given system. We utilize the publicly available Jama package [43] to compute each state-specific autoregressive mean.

Specific details of the estimation of initial state, transition and emission parameters of the autoregressive higher-order HMM and a computational scheme of the training algorithm are given in Appendix B in Text S1.

#### Model Initialization

For the identification of differentially expressed genes in tumors, an initial autoregressive HMM must be specified to enable a good adaptation of the model to the data during the training. We transfer initial model and prior settings described in [13] to autoregressive HMMs. The initial state probabilities are set to 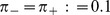 and 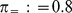 assuming that the proportion of differentially expressed genes is much less than the proportion of genes with unchanged expression behavior. We further assume that the initial transition matrix 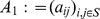 has a stationary distribution identical to the initial state distribution 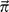. This is done by defining state-specific diagonal elements 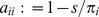 and non-diagonal elements 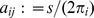 with respect to a scaling factor 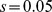 for controlling the state durations. Additionally, in analogy to [24], the transition probabilities of each transition matrix 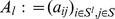 with 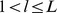 are initially set to 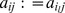 defining that the transition probability from each state-context 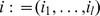 to a state 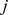 is identical to the value of the corresponding transition probability 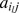 in 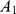. The initial state-specific autoregressive Gaussian emission densities are characterized by basic means 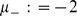, 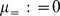 and 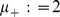 and corresponding standard deviations 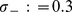, 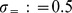 and 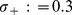. Additionally, we set each corresponding initial autoregressive coefficient 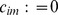. We also utilize these settings to specify appropriate prior knowledge for the training of the emission parameters of each state. Details of this are given in Appendix A in Text S1.

This basic initialization strategy has led to robust results in [13]. We here further performed an in-depth sensitivity analysis by systematically varying initial model and prior parameter settings to quantify the impact on the predictions made by autoregressive HMMs. We find that changes of parameter settings do not substantially influence the predictions (see results section for more details). Yet, users are still able to change these settings to enable modeling of specific characteristics of other data sets. However, this was not necessary for the analysis of the three different tumor data sets that we have analyzed here.

With the goal of performing exhaustive comparisons of different autoregressive HMMs, we trained each HMM with an autoregressive emission process of order 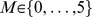 in combination with a state-transition process of order 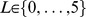 on the breast cancer expression profiles using the developed Bayesian Baum-Welch algorithm. This was done using the proposed initial basic settings. These settings were also used for the analysis of the glioma data sets.

## Results/Discussion

In this section, we first systematically compare different autoregressive HMMs for their performance to identify overexpressed genes with underlying increased copy numbers in breast cancer. Next, we analyze to which extent the obtained results can be transferred to other similar breast cancer gene expression profiles. This is complemented by performing a sensitivity analysis to study the robustness of predictions obtained by autoregressive HMMs. After that, we compare the performance of autoregressive HMMs to related existing methods. Here, we first investigate the different methods for general characteristics of their predictions of differentially expressed genes. We further compare the different methods based on their identification of overexpressed genes with underlying increased copy numbers in breast cancer. Finally, we transfer autoregressive higher-order HMMs to different types of gliomas and perform an in-depth systematic analysis of differentially expressed genes with respect to known cancer signaling pathways.

### Identification of Overexpressed Genes with Increased Gene Copy Numbers by Autoregressive Higher-Order Hidden Markov Models of Different Model Complexities

We utilize breast cancer gene expression and corresponding gene copy number data by *Pollack et al*. [3] to compare autoregressive HMMs with different combinations of emission and transition orders for their ability to predicted overexpressed genes with underlying increased copy numbers. This is generally motivated by a frequently observed direct genome-wide coupling between gene expression levels and gene copy numbers. Different studies comparing tumor to healthy tissue have found that genes with increased copy numbers tend to be overexpressed, while genes affected by deletions tend to be underexpressed in tumors (e.g. [3]–[6], [44], [45]). Since mutations in trans-acting factors (e.g. transcription factors or protein kinases) also lead to alterations of gene expression levels in tumor, there is of course not a direct one-to-one relationship between the expression level and the copy number of each gene. Still, genes affected by copy number alterations can be used to initially characterize and evaluate the identification of differentially expressed genes by different autoregressive HMMs.

Here, we do this in analogy to *Seifert et al*. [13] utilizing the strong coupling between overexpression and increased copy numbers of genes present in the breast cancer data set [3]. Based on that, we determined potential candidate genes of overexpression for each individual tumor sample using its corresponding individual gene copy number profile. For each individual tumor expression profile, each gene with an underlying increased gene copy number in tumor compared to normal was considered as a potential candidate for overexpression. We used three different copy number cutoffs (two-, three-, four-fold) for the selection of candidate genes of overexpression to evaluate the robustness of the model comparisons. The majority of these candidate genes tend to be overexpressed in breast cancer (Figure S3 in Text S1).

**Figure 3:**
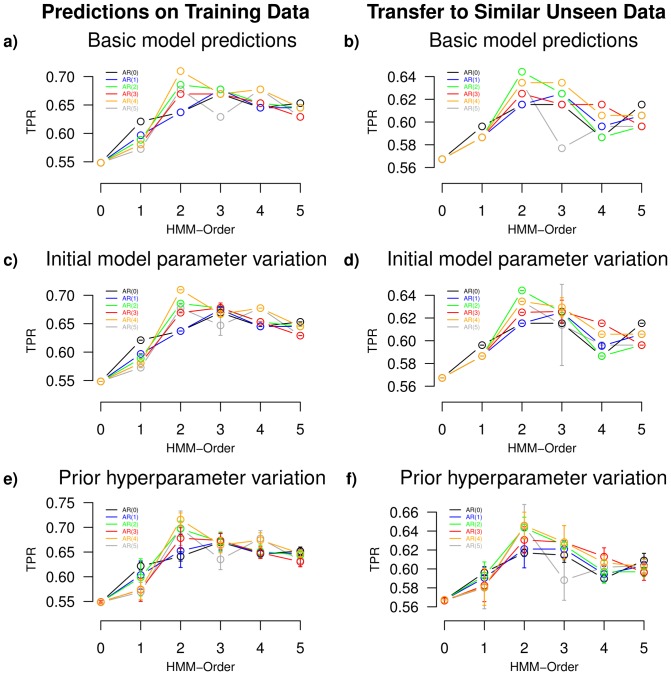
Identification of overexpressed genes with increased copy numbers in breast cancer by different autoregressive HMMs. Systematic comparison of the identification of overexpressed genes with at least three-fold increased copy numbers by autoregressive HMMs based on breast cancer gene expression profiles from [3]. Each AR(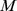)-HMM(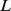) with an emission process of order 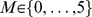 (AR(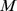)) in combination with a state-transition process of order 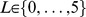 (HMM-Order) is considered. The left column shows the performances reached by autoregressive HMMs trained and applied to fifty percent of the breast cancer gene expression data. The right column shows the performances of these models reached on the remaining unseen fifty percent of the data set. For each model, the identification of candidate genes of overexpression with at least three-fold increased copy numbers is quantified by the true positive rate (TPR) reached at a fixed false positive rate of 5%. Six different scenarios are shown. **a**) and **b**) represent performances of the different models with respect to our standard initial model parameter settings. **c**) and **d**) represent average performances and corresponding standard deviations reached with respect to systematically changed initial model parameters. **e**) and **f**) represent average performances and corresponding standard deviations reached with respect to systematically modified prior hyperparameter settings. The predictions of autoregressive HMMs are generally very robust. Models utilizing a combination of higher-order state-transitions and autoregressive emissions (e.g. AR(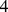)-HMM(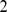) and AR(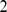)-HMM(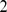)) are clearly outperforming the mixture model (AR(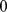)-HMM(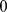)), the standard first-order HMM (AR(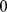)-HMM(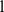)), and standard higher-order HMMs (AR(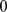)-HMM(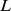)).

To analyze the performance of the identification of overexpressed genes with underlying increased copy numbers by different autoregressive HMMs, we used fifty percent of the breast cancer gene expression data set. We trained each initial AR(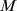)-HMM(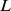) with an autoregressive emission process of order 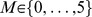 in combination with a state-transition process of order 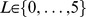 using the developed Bayesian Baum-Welch algorithm. We then compared the different models based on their performance to identify candidate genes of overexpression with at least three-fold increased copy numbers. To measure the performance of each individual model, we first ranked all genes according to their potential of being overexpressed utilizing the corresponding state-posteriors of state ‘
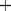
’. Based on that, we computed the true positive rate (TPR) of predicted potential candidate genes of overexpression reached at a typically considered fixed small false positive rate (FPR) of 5%. The reached performances are shown in Figure 3a. Similar results were reached considering the identification of candidate genes of overexpression with at least two- or four-fold increased copy numbers (Figure S4a in Text S1).

Generally, comparing well-known standard models included in the class of autoregressive higher-order HMMs, the mixture model (AR(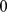)-HMM(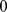)), which does not model dependencies between expression levels of genes, and the autoregressive mixture model (AR(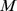)-HMM(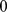) with 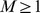), which directly models dependencies between expression levels of successive genes on chromosomes, are outperformed by non-autoregressive HMMs (AR(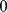)-HMM(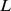) with 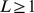: black curves in Figure 3a), which integrate dependencies between genes using state-transitions. This extends previous findings where standard mixture models have been outperformed by first-order and higher-order HMMs [11], [13], [24]. Moreover, these performances can be further improved by autoregressive higher-order HMMs (AR(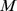)-HMM(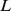) with 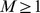 and 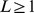; non-black curves in Figure 3a). Especially, autoregressive HMMs with an emission process of order two up to four in combination with a state-transition process of order two up to three reached the best performances.

In summary, autoregressive higher-order HMMs have the potential to improve the identification of candidate genes of overexpression with underlying increased gene copy numbers. However, this is not limited to the identification of overexpressed genes with underlying increased copy numbers in general. We have already previously shown on the same data set that HMMs with identical initial model parameters as considered here are valuable for the identification of hotspot genes of differential expression in breast cancer [13]. Thus, since the autoregressive HMMs considered here and the previously considered HMMs were very similar after training, this characteristic is also transferred to autoregressive HMMs.

### Transfer of Autoregressive Higher-Order Hidden Markov Models to Similar Breast Cancer Gene Expression Profiles

Next, we utilized the remaining fifty percent of the previously considered breast cancer gene expression data set to test the performance of the previously trained autoregressive HMMs on other similar but previously unseen gene expression profiles. Generally, we note that such a transfer is only convenient if both data sets have similar measurement characteristics (e.g. distribution of log-ratios, relatively similar expression profiles). This is also reflected in closely related array comparative genomic hybridization (aCGH) data analysis pipelines, where models are initially adapted and later used for the analysis of a single individual data set, but where transfers of trained models to other data sets are typically not done. However, a successful transfer of trained HMMs from one data set to a new data set can especially save time for the analysis of large data sets by avoiding time-consuming training steps. Thus, we transferred all previously trained autoregressive HMMs to other similar breast cancer gene expression profiles to evaluate the reached model performances after this transfer in analogy to our previous study.

The results are summarized in Figure 3b. Similar results were obtained for the identification of candidate genes of overexpression with at least two- or four-fold increased copy numbers (Figure S4b in Text S1). We find that the transfer of trained autoregressive HMMs to other similar breast cancer data leads to prediction characteristics (Figure 3b) that are comparable in shape with the predictions reached on the data initially used to train the models (Figure 3a). However, as expected, the transfer of models from the initially used training data to other independent tumor expression profiles leads to a reduction of the prediction performance (in average a reduction of 3.8 in TPR for HMMs excluding mixture models that gained 1.9 in TPR at a fixed FPR of 5%). Still, autoregressive higher-order HMMs outperform standard non-autoregressive first- and higher-order HMMs. Again, second-order HMMs with autoregressive emissions of order two or four (AR(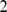)-HMM(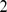), AR(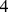)-HMM(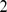)) are among the best models. Apart from that, the relatively unstable predictions of autoregressive HMMs with a fifth-order emission process (AR(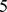)-HMM(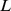) with 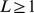) on the training data are also transferred to the test data (grey curves in Figure 3). Since both data sets have similar measurement distributions, this tends to reflect an overadaptation due to the large number of free emission parameters.

Thus, this study indicates that a careful transfer of previously trained autoregressive HMMs to other similar tumor expression profiles can be realized, but one should keep in mind that a faster analysis of large data sets may result in reduced accuracy of predictions.

### Analysis of the Robustness of Predictions Made by Autoregressive Higher-Order Hidden Markov Models

We additionally performed a sensitivity analysis by systematically varying initial model parameters and prior parameter settings to analyze the robustness of the predictions made by autoregressive HMMs. Since this is generally very time-consuming, we focused on those parameters that are expected to have the greatest influence on the prediction of differentially expressed genes. We first tested twelve different model initializations and trained corresponding autoregressive HMMs on fifty percent of the breast cancer gene expression data set using identical prior hyperparameter settings. Specific details are given in Table S1 in Text S1. We considered each AR(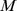)-HMM(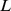) with an autoregressive emission process of order 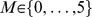 in combination with a state-transition process of order 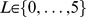 under consideration of clearly varying initial model parameters. In analogy to the two previous model evaluation studies, we evaluated the prediction of overexpressed genes with underlying increased gene copy numbers by the different trained autoregressive HMMs on the initially considered training data and on other similar breast cancer gene expression profiles. We generally find that the corresponding predictions of the different autoregressive HMMs are very stable (Figure 3c and d) and widely comparable to those reached by autoregressive HMMs utilizing our generally considered initial model parameterization specified in the methods section (Figure 3a and b). Similar results were reached considering the identification of overexpressed genes with at least two- or four-fold increased copy numbers (Figure S5 in Text S1).

We next tested twelve different prior parameter settings and trained the corresponding autoregressive HMMs utilizing our generally considered fixed initial model parameterization described in the methods section. Details of the tested prior parameter settings are provided in Table S1 in Text S1. Again, we find that the predictions of the corresponding autoregressive HMMs are very stable (Figure 3e and f). The predictions reached by autoregressive HMMs based on the twelve different prior parameter settings vary slightly more than those reached for the twelve different model initializations. Still, we clearly find that autoregressive HMMs have the potential to improve the predictions of overexpressed genes with underlying increased copy numbers compared to mixture models (AR(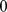)-HMM(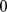)) and standard first- or higher-order HMMs (AR(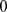)-HMM(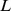) with 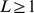). This is also transferred to predictions of overexpressed genes with at least two- or four-fold increased copy numbers (Figure S6 in Text S1).

In summary, this study indicates that changes of the initial model parameter settings and of the prior parameter settings do not substantially affect the predictions of autoregressive HMMs. Thus, the predictions of autoregressive HMMs are robust with respect to reasonable changes of initial parameter settings.

### Comparison of General Characteristics of the Identification of Differentially Expressed Genes by Autoregressive Higher-Order Hidden Markov Models to Related Methods

To compare the identification of differentially expressed genes by autoregressive HMMs to related methods, we analyzed the breast cancer gene expression data set [3] using the AR(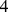)-HMM(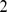) and nine other methods. We include ChARM [12], which has been demonstrated to work on aCGH and tumor expression profiles into this comparison. We also compare our model to MixMod and DSHMM, which both were specifically developed for the analysis of tumor expression profiles [13]. Additionally, we also include methods from the closely related field of aCGH analysis (Wavelet [46], BioHMM [47], FHMM [48], CBS [49], CGHseg [50], GLAD [51]) into this comparison. A more detailed summary of considered methods is given in Table S2 in Text S1.

We applied all methods with their proposed initial standard settings to all breast cancer gene expression profiles. We note that experts of specific methods might be able to further improve the predictions by fine-tuning of specific model parameters. The different methods implement specific algorithms to adapt their initial parameters or test statistics to the data followed by the prediction step. All methods except ChARM, DSHMM, AR(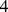)-HMM(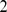) and MixMod were run on the ADaCGH web-server [52]. The ADaCGH output assigns one of the three labels ‘
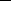
’, ‘
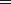
’, or ‘
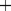
’ to each gene in a tumor expression profile. This can be directly interpreted as underexpressed, unchanged expressed, or overexpressed in tumor compared to healthy tissue. For ChARM, each non-significantly changed gene was labeled as unchanged expressed and each significantly changed gene was labeled either as underexpressed or overexpressed depending on the sign of the corresponding log-ratio. For DSHMM, AR(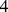)-HMM(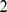) and MixMod, we utilize the corresponding state-posterior decoding to assign the most likely label to each gene. We further note that we utilized the same initial basic settings as reported in the method sections for DSHMM, AR(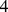)-HMM(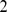) and MixMod to enable an unbiased direct comparison of these three methods.

To characterize the potential of the different methods to identify differentially expressed genes, we first determined for each method the proportions of predicted under- and overexpressed genes in relation to the total number of measured genes. Additionally, we computed the means and the standard deviations of measured log-ratios for genes predicted as under- and overexpressed by each method. Generally, the proportion of predictions quantifies the prediction behavior of each method, and this measure in combination with the mean and the standard deviation of the log-ratios allows to analyze the ability to predict differentially expressed genes. Finally, we also measured the runtime of each method required for the analysis of the breast cancer data set. The results are summarized in Table 1.

**Table 1:** Method-specific characteristics for the identification of differentially expressed genes in breast cancer.

		Underexpressed Genes			Overexpressed Genes			
Method	Reference	Prop.	Mean	Sd	Prop.	Mean	Sd	Runtime
Wavelet	[46]	18.58%	−0.09	0.63	9.52%	0.13	0.84	3 min 36 s
BioHMM	[47]	7.41%	−0.30	0.88	9.96%	0.39	0.90	5 min 03 s
FHMM	[48]	6.37%	−0.37	0.75	5.42%	0.62	0.92	2 min 59 s
CBS	[49]	2.66%	−0.19	0.72	1.91%	0.47	0.98	3 min 02 s
CGHseg	[50]	2.45%	−0.11	0.64	0.97%	0.33	1.10	2 min 52 s
ChARM	[12]	1.02%	−0.30	0.66	1.84%	0.31	0.77	-
GLAD	[51]	1.54%	−1.95	1.00	1.77%	1.85	0.76	2 min 51 s
DSHMM	[13]	1.48%	−2.18	0.97	2.25%	1.90	0.70	1 min 26 s
AR( 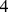 )-HMM( 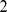 )	see Methods	1.51%	−2.23	0.88	2.19%	1.85	0.66	2 min 56 s
MixMod	[13]	1.34%	−2.39	0.81	1.84%	2.13	0.57	11 s

The proportion of genes predicted as under- or overexpressed in relation to the total number of measured genes and the corresponding means and standard deviations of the underlying measured log-ratios are summarized for each method based on the the breast cancer gene expression data set from [3]. The different methods were grouped into three categories according to their proportion of identified differentially expressed genes and the corresponding mean log-ratio columns. The rightmost column specifies the runtimes of the different methods required to analyze the data set. All methods except ChARM, MixMod, DSHMM and AR(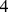)-HMM(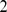) were run on the ADaCGH web-server [52] utilizing parallel computations (AMD Opteron 2.2 GHz CPUs with 6 GB RAM). The remaining methods were run on a standard laptop with Intel CPU T9500 2.6 GHz and 2 GB RAM.

We generally observe that the methods can be grouped into three classes. The first class represents Wavelet, BioHMM and FHMM, which all predict a much larger proportion of underexpressed and overexpressed genes than all other methods. However, for these methods, the means and the standard deviations of the corresponding log-ratios show that a large proportion of predicted differentially expressed genes have log-ratios that are close to zero. Additionally, genes predicted as underexpressed can have log-ratios greater than zero. Vice versa, genes predicted as overexpressed can have log-ratios less than zero. This does not represent a solid identification of differentially expressed genes, because underexpressed genes are expected to have log-ratios much less than zero and overexpressed genes are expected to have log-ratios much greater than zero. Thus, these methods should not be transferred to the analysis of tumor gene expression data. The second class consists of CBS, CGHseg and ChARM that all predict much less underexpressed and overexpressed genes than the methods of the first class. However, still the same problems as observed for methods from the first class occur. The methods represented by the third class are GLAD, DSHMM, AR(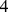)-HMM(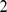) and MixMod. These four methods also predict a much smaller number of genes as underexpressed and overexpressed than methods of the first class. Additionally, the predicted underexpressed and overexpressed genes reflect corresponding characteristic log-ratios as expected for differentially expressed genes. That is, genes predicted as underexpressed have log-ratios clearly less than zero and genes predicted as overexpressed have log-ratios clearly greater than zero. Thus, the autoregressive higher-order HMM and the three other methods reach a solid identification of differentially expressed genes in individual tumor expression profiles. The runtimes of these methods were all less than three minutes. Since the standard mixture model (MixMod) does not model chromosomal dependencies between adjacent genes, it was extremely fast requiring only 11 seconds. However, the AR(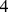)-HMM(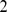) with a fourth-order emission process and a second-order state-transition process had nearly the same runtime as GLAD, which was run on the ADaCGH web-server utilizing parallel computations. To further evaluate all different methods, we subsequently study how the more complex modeling of local chromosomal dependencies by autoregressive higher-order HMMs influences the predictions of overexpressed genes with known underlying increased copy numbers in comparison to the other methods.

### Comparison of the Identification of Overexpressed Genes with Increased Copy Numbers by Autoregressive Higher-Order Hidden Markov Models to Related Methods

**Figure 4:**
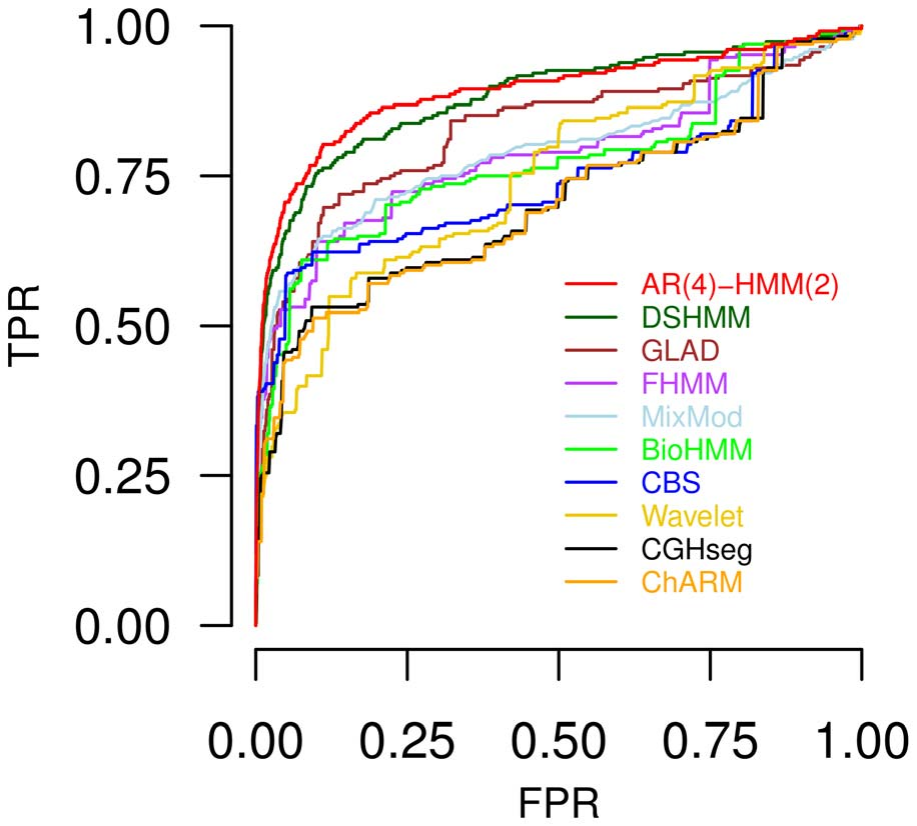
Comparison of an autoregressive higher-order HMM to related existing methods. Comparison of the AR(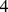)-HMM(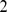) and related methods with respect to the identification of overexpressed genes with at least three-fold increased copy numbers based on breast cancer data from [3]. The performance of each method is quantified by a receiver operating characteristic (ROC) curve displaying the true positive rate (TPR) reached at different levels of false positive rates (FPR). The AR(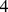)-HMM(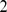) with a fourth-order autoregressive emission process and a second-order state-transition process reaches the best performance (red).

We next exemplarily compare the identification of potential candidate genes of overexpression with underlying increased gene copy numbers by the AR(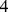)-HMM(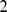) to all nine previously considered methods. Especially, the comparison to the DSHMM, a first-order HMM that integrates spatial distances of adjacent genes into the transition process, is of great interest, because this model has previously been identified to reach the best performance in a similar study [13]. We utilized the predictions from the previous sections to rank the genes according to their decreasing potential of being overexpressed. Due to the lack of method-specific scores for performing rankings of genes for methods run on the ADaCGH web-server and ChARM, we computed average segmental log-ratios based on the corresponding discrete segmentations returned for each individual tumor expression profile. Based on that, we assigned the corresponding average segmental log-ratio to each gene enabling us to rank the genes according to their potential of being overexpressed. For AR(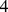)-HMM(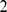), DSHMM, and MixMod, we utilized the state-posteriors of state ‘
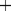
’ modeling overexpressed genes to rank the predictions. Based on that, we evaluated the prediction of candidate genes of overexpression with at least two-, three- or four-fold increased copy numbers in the breast cancer data set [3] at different levels of false positives. The corresponding receiver operating characteristic (ROC) curves for predicted overexpressed genes with at least three-fold increased copy numbers are shown in Figure 4. Very similar ROC curves were obtained for the prediction of overexpressed genes with at least two- or four-fold increased copy numbers (Figure S7 in Text S1). An additional comprehensive summary for true positive rates reached at small fixed false positive rates and globally reached areas under the ROC curves are given in Table S3 in Text S1. Generally, we find that the AR(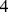)-HMM(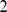) outperforms all methods including the DSHMM previously identified as the best performer [13]. This performance benefit can be attributed to the more complex modeling of spatial dependencies between genes by utilizing a higher-order state-transition process in combination with an autoregressive emission process. That this more complex modeling leads to a gain in performance is clearly shown by the comparison of the AR(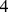)-HMM(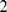) to the mixture model (MixMod), which does not model dependencies between genes (see Figure 2 for underlying state space representation), and by the comparison to the DSHMM, which specifically models dependencies between directly adjacent genes on a chromosome.

Together with the previous finding that autoregressive HMMs can identify differentially expressed genes independent of underlying gene copy numbers (Table 1), this analysis further supports that autoregressive HMMs are useful for the identification of differentially expressed genes in tumors.

### Application of Autoregressive Higher-Order Hidden Markov Models to Glioma Data

Finally, we apply our autoregressive higher-order HMMs to different case studies utilizing publicly available glioma gene expression data sets. Here, we exemplarily report the most striking findings concerning the systematic analysis of gene expression changes in known cancer signaling pathways. The other case studies focusing on the identification of hotspots of differential expression in glioblastomas based on a data set by *de Tayrac* [4] and corresponding expression patterns of hotspot genes in different types of gliomas from the Rembrandt repository are summarized in Appendix C in Text S1. Briefly, we were able to identify seven novel previously unreported hotspots of differential expression in glioblastomas that we discuss together with other interesting hotspot genes identified under less stringent hotspot criteria. In the following, we focus on the in-depth analysis of the predictions obtained for different types of gliomas with respect to known cancer signaling pathways.

#### Systematic Analysis of Gene Expression Changes in Different Types of Gliomas With Respect to Known Cancer Signaling Pathways

Here, we investigate the potential of autoregressive higher-order HMMs to identify alterations of expression levels of genes in known cancer signaling pathways for different types of gliomas (oligodendrogliomas, astrocytomas, glioblastomas). The classification of gliomas according to these subtypes has important implications for the prognosis and the treatment of glioma patients [53]. However, differences in gene expression levels of known cancer signaling pathways are still poorly understood and not well characterized. We therefore adapted the AR(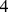)-HMM(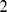) based on the initial standard settings described in the methods section to the average expression profile of gliomas in the Rembrandt repository and used the obtained model to predict the expression behavior in each individual glioma sample. This prediction was done by computing the state-posterior probabilities of the states ‘
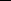
’ and ‘
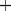
’ under the AR(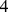)-HMM(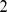) for each individual gene in each individual tumor to obtain a measure that enables to distinguish how likely it is that a specific gene is underexpressed or overexpressed in tumor compared to the normal brain reference. We now summed these gene-specific probability scores of state ‘
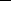
’ and ‘
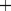
’ across all oligodendrogliomas, astrocytomas and glioblastomas separately to obtain ranking lists of top candidate genes of under- and overexpression for the three different types of gliomas. We then analyzed the overlap of the resulting top genes of under- and overexpression and known cancer signaling pathways based on the KEGG pathways in cancer overview (path:hsa05200) for which we compiled corresponding pathway genes based on ConsensusPathDB [54]. We further extended the initial KEGG cancer pathway view by systematically adding other known cancer-relevant pathways for DNA repair, Telomere maintenance, DNA replication and Hedgehog signaling (see Table S4 in Text S1 for an overview with functional annotations). Based on this, we systematically considered increasing numbers of top ranking genes of under- and overexpression and determined the corresponding numbers of genes that overlap with the individual cancer signaling pathways.

A representative global overview of how strongly oligodendrogliomas, astrocytomas and glioblastomas are affected by under- and overexpression in specific cancer signaling pathways with respect to random expectations is shown for the top 300 genes in Figure S8 in Text S1. Globally looking at all genes in pathways together, we find a significant enrichment of top-ranking overexpressed genes across all known cancer signaling pathways for each type of glioma. These enrichments were at least twice as much as randomly expected (90 genes in oligodendrogliomas, 91 genes in astrocytomas and 102 genes in glioblastomas compared to 45 expected by chance at the level of the top 300). We do not observe such global trends across all known cancer signaling pathways for the top-ranking underexpressed genes. Each type of glioma showed nearly the same number of underexpressed genes than randomly expected (48 genes in oligodendrogliomas, 50 genes in astrocytomas and 47 genes in glioblastomas compared to 45 expected by chance at the level of the top 300).

In more detail, considering underexpressed genes, we observe that the same cancer signaling pathways are always affected by nearly the same number of genes independent of the type of glioma (Figure S8a in Text S1). In comparison to random expectations, the pathways that are most strongly affected by underexpression are MAPK, ErbB, TGF-Beta and VEGF signaling. These pathways play important roles in the regulation of cell proliferation, differentiation, migration, survival, adhesion, apoptosis and angiogenesis. It is also interesting to note that we do not observe underexpressed genes in p53 signaling, Notch signaling, and Hedgehog signaling, Cytokine-cytokine receptor interaction, DNA replication, DNA repair, and Telomere maintenance across the three different types of gliomas.

In contrast to this, these pathway-based characteristics are largely different considering the top 300 overexpressed genes (Figure S8b in Text S1). The most homogeneous behavior in terms of number of overexpressed genes across the three different types of gliomas is observed for the cell cycle, Wnt signaling and p53 signaling pathways. The Wnt pathway plays important roles in cell polarization, proliferation, differentiation and migration, whereas the p53 pathway has a central role in cellular signaling with major functions in controlling apoptosis, cellular senescence and cell cycle arrest. Dysregulation of both signaling pathways is commonly found in various cancers [55], [56]. Interestingly, in addition to the homogeneous behavior of these three pathways, we also observe significant differences between oligodendrogliomas, astrocytomas and glioblastomas at the level of the top 300 overexpressed genes that we analyze in the following.

**Figure 5:**
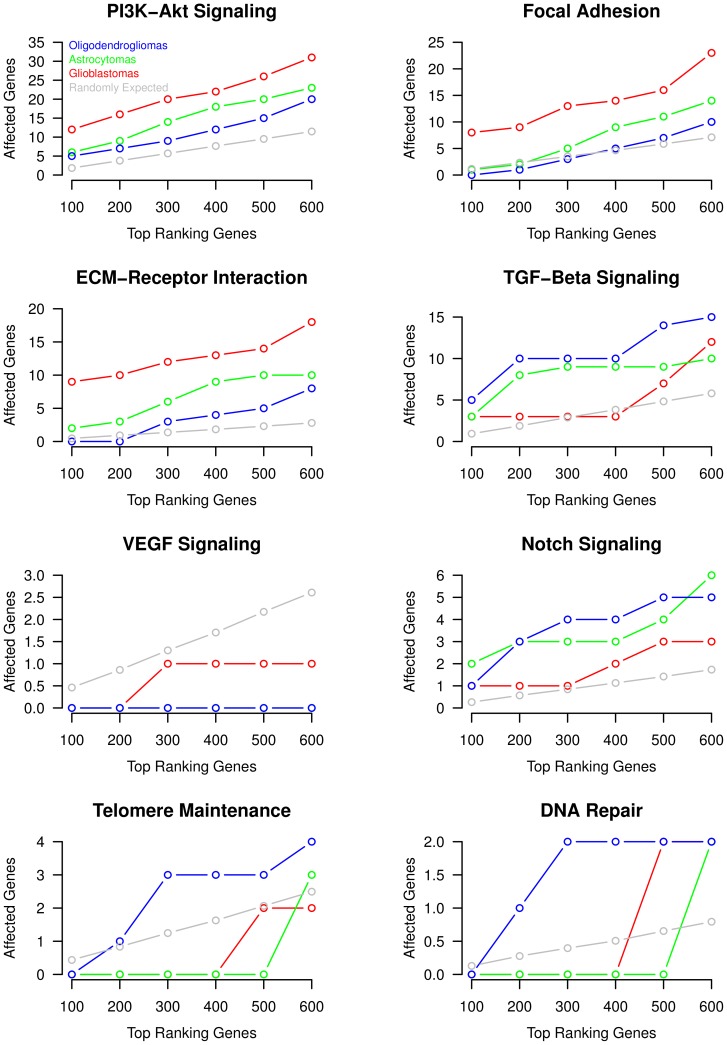
Most discriminative signaling pathways distinguishing different types of gliomas. Overview of known cancer signaling pathways identified to show largely distinct patterns of overexpressed genes in oligodendrogliomas, astrocytomas and glioblastomas based on predictions of the AR(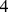)-HMM(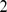). The overlap of the top-ranking overexpressed genes with the specific signaling pathways is quantified from top 100 to top 600. Grey curves show random expectations with respect to the number of genes in a specific pathway. Robust systematic differences between the different types of gliomas are clearly visible.

Considering increasing numbers of top-ranking overexpressed genes, we observe robust systematic differences between oligodendrogliomas, astrocytomas and glioblastomas for PI3K-Akt signaling, Focal adhesion, ECM-Receptor interaction, TGF-Beta signaling, VEGF signaling, Notch signaling, Telomere maintenance and DNA repair (Figure 5). Glioblastomas show clearly more overexpressed genes in PI3K-Akt signaling, Focal adhesion and ECM-Receptor interaction than astrocytomas, which show a greater number of overexpressed genes in these pathways than oligodendrogliomas. Since these pathways are known to be involved in cell survival, proliferation, migration and metabolism, a systematic increase in overexpressed genes may lead to increased pathway activities contributing to the generally observed increased malignancy of glioblastomas in comparison to astrocytomas and oligodendrogliomas [53]. Interestingly, we observe an inverse behavior for TGF-Beta signaling, Notch signaling, Telomere maintenance, and DNA repair (Figure 5). For these pathways, oligodendrogliomas show the greatest number of overexpressed genes followed by astrocytomas, which tend to have a greater number of overexpressed genes than glioblastomas. Since TGF-Beta signaling is involved in apoptosis and cell cycle arrest and since an increased expression of Telomere maintenance and DNA repair pathways may contribute to more stable tumor genomes, this increased number of overexpressed genes observed for oligodendrogliomas may be associated with the generally better prognosis of oligodendrogliomas in comparison to astrocytomas and glioblastomas [53]. These observations further sharpen and support initial findings that expression profiles of oligodendrogliomas are enriched in overexpressed genes involved in DNA repair in a study comparing oligodendrogliomas, astrocytomas and glioblastomas [57]. Additionally, also the revealed over-representation of the Notch pathway in oligodendrogliomas and astrocytomas compared to glioblastomas is very interesting. Differences in the activities of the Notch pathway have been observed between astrocytomas and glioblastomas [58]. Moreover, the Notch pathway has been reported to mediate opposite effects either acting in an oncogenic or tumor-suppressive manner in dependency of the different Notch trans-membrane receptors [58]. While Notch-1 was found to be inhibitory for tumor growth, Notch-2 was found to promote tumorgenesis in medulloblastoma [59]. Comparing the rankings of Notch-1 and Notch-2 in our study, we find that Notch-1 but not Notch-2 is strongly overexpressed in oligodendrogliomas (rank 108 for Notch-1 vs. 2316 for Notch-2). This indicates that the characteristic overexpression of Notch-1 as part of the Notch pathway may contribute to the better prognosis of oligodendrogliomas [53]. This is further supported by the observation that Notch-1 is an important hub gene in proneural oligodendrogliomas, which show improved survival among different types of gliomas [60]. Indeed, except for four oligodendrogliomas, we find that all our oligodendrogliomas are classified as proneural utilizing subtype-specific gene expression classification signatures from [61]. Thus, our findings further strengthen the important role of the Notch signaling pathway in oligodendrogliomas. In addition to this, another important finding is that the VEGF pathway is only affected in glioblastomas by the overexpression of the VEGFA gene. This is in accordance with generally observed histological characteristics of glioblastomas that are highly angiogenic and characterized by the presence of vascular proliferations [62].

**Figure 6:**
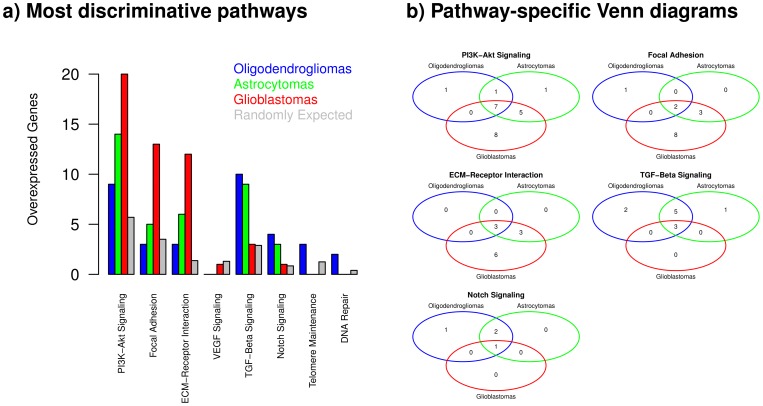
Systematic characterization of the most discriminative signaling pathways distinguishing different types of gliomas. **a**) Characteristic view on the most discriminative pathways between oligodendrogliomas, astrocytomas and glioblastomas at the level of the top 300 overexpressed genes in Figure 5. **b**) Selected gene-based view on the most discriminative signaling pathways shown in a). The Venn diagrams show pathway-specific overlaps of overexpressed genes between the different types of gliomas. The strong overlap of genes between the different types of gliomas indicates the presence of common core sets of affected genes. These pathway-specific core gene sets are further extended towards the glioma with the greatest number of overexpressed genes. The corresponding genes are summarized in Table 2.

Finally, we analyze the most discriminative pathways between oligodendrogliomas, astrocytomas and glioblastomas shown in Figure 5 at the level of single genes to investigate whether the different types of gliomas utilize the same sets of genes to alter pathway activities. Corresponding pathway-specific barplots and Venn diagrams for the top 300 overexpressed genes are shown in Figure 6. The Venn diagrams clearly indicate that the different types of gliomas mainly utilize pathway-specific common core sets of affected genes. These pathway-specific core gene sets are further extended towards the type of glioma with the greatest number of overexpressed genes. Since this observation might be of potential relevance for the development of tumor-specific markers and future treatment strategies, we have summarized the underlying genes and their corresponding pathway memberships in the most discriminative cancer signaling pathways in Table 2. Interestingly, it is important to note that of the 41 listed genes 17 genes are playing a role in at least two pathways and that 12 of these genes are involved in three pathways. Among the genes involved in three pathways, the combination of ECM-Receptor interaction, PI3K-Akt signaling and Focal adhesion is strongly overrepresented, which underlines the complexity and the interplay of pathway alterations in tumors.

**Table 2:** Genes overexpressed in the most discriminative pathways distinguishing different types of gliomas.

Gene	OD	AS	GBM	Signaling Pathways	Annotation
ANGPT2	0	0	1	PI3K-Akt	Angiopoietin-2
CAV1	0	0	1	Focal Adh.	Caveolin
COL1A1	0	0	1	ECM, Focal Adh., PI3K-Akt	Collagen alpha-1(I) chain
COL4A2	0	0	1	ECM, Focal Adh., PI3K-Akt	Canstatin
COL5A2	0	0	1	ECM, Focal Adh., PI3K-Akt	Collagen alpha-2(V) chain
FN1	0	0	1	ECM, Focal Adh., PI3K-Akt	Ugl-Y3
LAMB1	0	0	1	ECM, Focal Adh., PI3K-Akt	Laminin subunit beta-1
LAMC1	0	0	1	ECM, Focal Adh., PI3K-Akt	Laminin subunit gamma-1
VEGFA	0	0	1	Focal Adh., PI3K-Akt, VEGF	Vascular endothelial growth factor A
RELA	0	1	0	PI3K-Akt	Transcription factor p65
TGFB1	0	1	0	TGF-Beta	Transforming growth factor beta-1
CDK2	0	1	1	PI3K-Akt	Cyclin-dependent kinase 2
COL1A2	0	1	1	ECM, Focal Adh., PI3K-Akt	Collagen alpha-2(I) chain
COL4A1	0	1	1	ECM, Focal Adh., PI3K-Akt	Collagen alpha-1(IV) chain
LAMB2	0	1	1	ECM, Focal Adh., PI3K-Akt	Laminin subunit gamma-1
TLR2	0	1	1	PI3K-Akt	Toll-like receptor 2
BMP7	1	0	0	TGF-Beta	Bone morphogenetic protein 7
NOG	1	0	0	TGF-Beta	Noggin
NOTCH1	1	0	0	Notch	Neurogenic locus notch homolog protein 1
PGF	1	0	0	Focal Adh., PI3K-Akt	Placenta growth factor
RFC4	1	0	0	DNA Repair, Telomere	Replication factor C subunit 4
RFC5	1	0	0	DNA Repair, Telomere	Replication factor C subunit 5
RUVBL1	1	0	0	Telomere	RuvB-like 1
BMP2	1	1	0	TGF-Beta	Bone morphogenetic protein 2
CBLB	1	1	0	TGF-Beta	E3 ubiquitin-protein ligase CBL-B
DDIT4	1	1	0	PI3K-Akt	DNA damage-inducible transcript 4 protein
DLL3	1	1	0	Notch	Delta-like protein 3
E2F5	1	1	0	TGF-Beta	Transcription factor E2F5
ID1	1	1	0	TGF-Beta	DNA-binding protein inhibitor ID-1
ID4	1	1	0	TGF-Beta	DNA-binding protein inhibitor ID-4
MAML2	1	1	0	Notch	Mastermind-like protein 2
CD44	1	1	1	ECM	CD44 antigen
CDK4	1	1	1	PI3K-Akt	Highly similar to Cell division protein kinase 4
COL3A1	1	1	1	ECM, Focal Adh., PI3K-Akt	Collagen alpha-1(III) chain
DTX3L	1	1	1	Notch	E3 ubiquitin-protein ligase DTX3L
EIF4EBP1	1	1	1	PI3K-Akt, TGF-Beta	Euk. transl. initiation factor 4E-binding protein 1
F2R	1	1	1	PI3K-Akt	Proteinase-activated receptor 1
ID3	1	1	1	TGF-Beta	DNA-binding protein inhibitor ID-3
MYC	1	1	1	PI3K-Akt, TGF-Beta	Myc proto-oncogene protein
TNC	1	1	1	ECM, Focal Adh., PI3K-Akt	Tenascin
TP53	1	1	1	PI3K-Akt	Cellular tumor antigen p53

Overexpressed genes representing the most discriminative cancer signaling pathways distinguishing oligodendrogliomas (OD), astrocytomas (AS) and glioblastomas (GBM) at the level of the top 300 genes (Figure 6). Genes identified as overexpressed in a specific glioma type are indicated by ‘1’, otherwise ‘0’. The column ‘Signaling Pathways’ represents the corresponding membership of each gene in one or more of these pathways.

In summary, we successfully applied our autoregressive higher-order HMM to the analysis of glioma gene expression profiles. We were able to identify known important cancer signaling pathway alterations, and we revealed systematic pathway-specific differences between different types of gliomas that may contribute to improve the understanding of glioma development.

### Conclusions

We have developed the novel model class of autoregressive higher-order HMMs that utilize local chromosomal dependencies of gene expression levels to improve the analysis of individual tumor expression profiles. Autoregressive higher-order HMMs form a very general model class that includes several well-known standard models such as mixture models, autoregressive mixture models, standard first-order HMMs, and standard higher-order HMMs as special cases. Based on in-depth comparison studies on breast cancer gene expression data, we have identified that autoregressive higher-order HMMs robustly predict overexpressed genes with known underlying increased copy numbers. These results were also widely confirmed when we transferred trained models to other similar but previously unseen breast cancer gene expression profiles. We have further identified that autoregressive HMMs are able to reach a solid characterization of differentially expressed genes independent of the underlying copy number status. Additional comparisons to closely related methods clearly indicate that autoregressive higher-order HMMs are very useful for the analysis of tumor expression profiles. This is also further supported by the identification of known and previously uncharacterized hotspots of differential expression in glioblastomas (see Appendix C in Text S1 for details).

Moreover, we were able to reveal novel interesting insights on the alterations of expression levels in known cancer signaling pathways across different types of gliomas by utilizing autoregressive higher-order HMMs. We identified characteristic pathway-specific expression patterns distinguishing oligodendrogliomas, astrocytomas and glioblastomas. Gene-based views on these pathways clearly indicate the presence of common core sets of genes that are jointly altered in these gliomas and further extended towards the specific type of glioma with the greatest number of affected pathway-specific genes. Additionally, a large fraction of these genes is involved in more than one pathway highlighting the complexity and the interplay of affected signaling mechanisms contributing to the development of gliomas. Our observations may therefore have important implications for the development of future treatment strategies.

Generally, the increase in performance reached by autoregressive higher-order HMMs can be attributed to the more complex modeling of local chromosomal dependencies between neighboring genes by utilizing a combination of higher-order state-transitions and autoregressive emissions. The separate usage of either (i) only higher-order state-transitions or (ii) only autoregressive emissions did not lead to performances reached by the best-performing autoregressive HMMs that combine these two features simultaneously. The best-performing models had state-transitions of order two to three in combination with autoregressive emissions of order two to four. For the analysis of other data sets, we suggest to consider the most parsimonious model of the best-performing models. That is, one may use the HMM with second-order state-transitions and second-order autoregressive emissions. Since this only involves second-order state-transitions, such a model can also be trained within reasonable time on large data sets. Altogether, our results clearly indicate that autoregressive higher-order HMMs are valuable tools for the analysis of individual tumor expression profiles.

Currently, autoregressive higher-order HMMs enable to analyze expression levels of genes in tumor with respect to their linear chromosomal order. This linear processing of gene expression levels allows to improve the predictions via the modeling of existing local chromosomal dependencies. This provides a solid basis that could be further extended by taking prior knowledge about underlying transcriptional networks (e.g. [63]) or spatial genome organization (e.g. [64]) into account.

## Supporting Information

Text S1 **Mathematical basics of the prior distributions for initial state, state-transition and emission parameters and details of the chosen prior hyperparameters are given in the section 'Appendix A: Prior distributions’.** A detailed derivation of the Bayesian Baum-Welch algorithm for autoregressive higher-order HMMs is given in the section ‘Appendix B: Bayesian Baum-Welch algorithm’. An in-depth analysis of identified hotspots of differential expression in gliomas is given in the section ‘Appendix C: Application of Autoregressive Higher-Order Hidden Markov Models to glioma data’. The supporting Figures S1–S8 are provided in the section ‘Supporting Figures’. The supporting Tables S1–S4 are given in the section ‘Supporting Tables’.
(PDF)
