# Supplementary material for: Autoregressive Higher-Order Hidden Markov Models: Exploiting Local Chromosomal Dependencies in the Analysis of Tumor Expression Profiles
Source: PLoS One. 2014 Jun 23;9(6):e100295. doi: 10.1371/journal.pone.0100295 (PMC4067306; doi:10.1371/journal.pone.0100295)
Supplement: Text S1 — Mathematical basics of the prior distributions for initial state, state-transition and emission parameters and details of the chosen prior hyperparameters are given in the section 'Appendix A: Prior distributions’. A detailed derivation of the Bayesian Baum-Welch algorithm for autoregressive higher-order HMMs is given in the section ‘Appendix B: Bayesian Baum-Welch algorithm’. An in-depth analysis of identified hotspots of differential expression in gliomas is given in the section ‘Appendix C: Application of Autoregressive Higher-Order Hidden Markov Models to glioma data’. The supporting Figures S1–S8 are provided in the section ‘Supporting Figures’. The supporting Tables S1–S4 are given in the section ‘Supporting Tables’. (PDF) [file pone.0100295.s001.pdf]

Supporting Information – Text S1

Autoregressive Higher-Order Hidden Markov Models: Exploiting Local Chromosomal Dependencies in the Analysis of Tumor Expression Profiles

Michael Seifert [1], Khalil Abou-El-Ardat [2], Betty Friedrich [1], Barbara Klink [2] and Andreas Deutsch [1]

[1]Innovative Methods of Computing, Center for Information Services and High Performance Computing, Dresden University of Technology, Dresden, Germany; [2]Institute for Clinical Genetics, Faculty of Medicine Carl Gustav Carus, Dresden University of Technology, Dresden, Germany

Contact: michael.seifert@zih.tu-dresden.de

Contents

1 Appendix A: Prior distributions 3

1.1 Prior for initial state distribution 3
1.2 Prior for set of transition matrices 3
1.3 Prior for emission parameters 4
1.4 Prior hyperparameters 5

2 Appendix B: Bayesian Baum-Welch algorithm 5

2.1 Basics of the Bayesian Baum-Welch algorithm 5
2.2 Splitting of Baum's auxiliary function 5
2.3 Estimation of initial state parameters 6
2.4 Estimation of transition parameters 7
2.5 Estimation of emission parameters 9
2.5.1 Baum's auxiliary function for emission parameters 9
2.5.2 Estimation of state-specific autoregressive means 10
2.5.3 Estimation of state-specific standard deviations 11
2.6 Computational scheme of the Bayesian Baum-Welch algorithm 11

3 Appendix C: Application of Autoregressive Higher-Order Hidden Markov Models to glioma data 12

3.1 Glioma data 12
3.2 Identification of hotspots of differential expression in glioblastomas 13
3.3 Analysis of identified hotspots of differential expression in the context of different types of gliomas 14

4 Supporting Figures **15**
4.1 Fig. S1: Histogram of log-ratios of the breast cancer gene expression data 15
4.2 Fig. S2: Three-state architecture of an AR-HMM 15
4.3 Fig. S3: Impact of increased gene copy numbers on gene expression levels . . . 16
4.4 Fig. S4: Identification of overexpressed genes with known increased copy numbers by different AR-HMMs 17
4.5 Fig. S5: Influence of varying initial model parameter settings on the identification of overexpressed genes with known increased copy numbers by different AR-HMMs 18
4.6 Fig. S6: Influence of varying prior hyperparameter settings on the identification of overexpressed genes with known increased copy numbers by different AR-HMMs 19
4.7 Fig. S7: ROC curves comparing AR-HMMs to existing methods 20
4.8 Fig. S8: Pathway-based view of differentially expressed genes in the context of different Rembrandt gliomas 21
4.9 Fig. S9: Hotspots of differential expression in glioblastomas 22
4.10 Fig. S10: Identified hotspots of differential expression in the context of different Rembrandt gliomas 23

5 Supporting Tables **25**
5.1 Tab. S1: Sensitivity analysis settings 25
5.2 Tab. S2: Summary of tested related methods 26
5.3 Tab. S3: Global performance comparison of different methods 27
5.4 Tab. S4: Known cancer signaling pathways 28
5.5 Tab. S5: Hotspots of overexpression in glioblastomas 29
5.6 Tab. S6: Hotspots of underexpression in glioblastomas 30
5.7 Tab. S7: Novel hotspots of underexpression in glioblastomas 31

1 Appendix A: Prior distributions

A problem-specific characterization of an autoregressive higher-order HMM is obtained by including prior knowledge about the distribution of measurements into the training of the model. This prior knowledge is integrated by defining a prior distribution

$$P[\,\lambda\,|\,\Theta\,] := D_1(\,\vec{\pi}\,|\,\Theta_1\,) \cdot D_2(\,A\,|\,\Theta_2\,) \cdot D_3(\,B\,|\,\Theta_3\,) \tag{1}$$

over the parameters of the HMM $\lambda := (\vec{\pi}, A, B)$. As outlined in the main manuscript, the prior distribution $P[\,\lambda\,|\,\Theta\,]$ is defined to be a product of independent prior distributions for the initial state distribution $\vec{\pi}$, the set of transition matrices A and the emission parameters B of the HMM.

1.1 Prior for initial state distribution

The conjugate prior for the initial state distribution $\vec{\pi} := (\pi_i)_{i \in S}$ with initial state probability $\pi_i := \exp(\Lambda_{\pi_i})$ is given by the transformed Dirichlet distribution

$$D_1(\,\vec{\pi}\,|\,\Theta_1\,) := Z(\Theta_1) \prod_{i \in S} \exp(\Lambda_{\pi_i} \cdot \vartheta_i) \tag{2}$$

with hyperparameter vector $\Theta_1 := (\vartheta_i)_{i \in S}$ that contains each pseudocount $\vartheta_i \in \mathbb{R}^+$. $Z(\Theta_1) := \Gamma(\sum_{i \in S} \vartheta_i) / \prod_{i \in S} \Gamma(\vartheta_i)$ with Gamma function $\Gamma(x) = \int_0^\infty u^{x-1} \cdot \exp(-u)\, du$ for all $x \in \mathbb{R}^+$ represents the normalization constant. The transformed Dirichlet prior has been specified in a general manner by MacKay (1998). Dirichlet priors are generally applied to model prior knowledge for initial state distributions (e.g. Durbin et al. (1998); Bishop (2006)). Following Seifert et al. (2011), each ϑ_i is set to $\vartheta_i = 3$.

1.2 Prior for set of transition matrices

The prior distribution for the set of transition matrices $A := \{A_1, \ldots, A_L\}$ is defined by a product of prior distributions

$$D_2(\,A\,|\,\Theta_2\,) := \prod_{l=1}^{L} D_2^l(\,A_l\,|\,\Theta_2^l\,)$$

under consideration of the hyperparameters $\Theta_2 := (\Theta_2^l)_{1 \le l \le L}$ and a prior distribution $D_2^l(\,A_l\,|\,\Theta_2^l\,)$ for each transition matrix $A_l \in A$. The prior for the transition matrix $A_l := (a_{ij})_{i \in S^l, j \in S}$ with transition probability $a_{ij} := \exp(\Lambda_{a_{ij}})$ is defined by a product of transformed Dirichlet distributions

$$D_2^l(\,A_l\,|\,\Theta_2^l\,) := \prod_{i \in S^l} Z(\Theta_{2,i}^l) \prod_{j \in S} \exp(\Lambda_{a_{ij}} \cdot \vartheta_{ij}) \tag{3}$$

in dependency of the hyperparameters $\Theta_2^l := (\Theta_{2,i}^l)_{i \in S^l}$ with hyperparameter vector $\Theta_{2,i}^l := (\vartheta_{ij})_{j \in S}$ containing each pseudocount $\vartheta_{ij} \in \mathbb{R}^+$. Each transformed Dirichlet distribution represents the specific conjugate prior for the state-specific transition probabilities of the corresponding state-context i of length l. The normalization constant $Z(.)$ is defined in analogy to

the previous section. Each ϑ_{ij} has been set to $\vartheta_{ij} = 3$.

1.3 Prior for emission parameters

In the following, we focus on the prior distribution $D_3(\, B \,|\, \Theta_3 \,)$ for the emission parameters to enable the integration of prior knowledge into the training of the state-specific autoregressive Gaussian emission densities. To avoid that each gene t in a tumor expression profile k is having its specific emission prior, we define the prior distribution for the state-specific autoregressive mean $\mu_i^t(k) := \mu_i + \sum_{m=1}^{M} c_{im} \cdot \vec{o}_{t-m}(k) \cdot \delta_{t,m}$ with $\delta_{t,m} = 0$ if $t - m < 1$ and otherwise $\delta_{t,m} = 1$ using the following strategy. We only explicitly model prior knowledge for each basic state-specific mean μ_i and assume constant priors for the corresponding coefficients $(c_{i1}, \ldots, c_{iM})$. This leads to a reduction of the prior for $\mu_i^t(k)$ to a prior that only depends on the corresponding μ_i. Based on that, the emission prior for an HMM with autoregressive Gaussian emissions can be defined by a product of independent Gaussian-Inverted-Gamma distributions as done for standard non-autoregressive HMMs in Seifert et al. (2011). Details of this emission prior in the context of autoregressive HMMs are considered subsequently.

The prior for the emission parameters B is defined by a product of independent Gaussian-Inverted-Gamma distributions

$$D_3(\, B \,|\, \Theta_3 \,) := \prod_{i \in S} N(\, \mu_i \,|\, \Theta_3 \,) \cdot I_G(\, \sigma_i \,|\, \Theta_3 \,) \tag{4}$$

with respect to the hyperparameter matrix $\Theta_3 := (\eta_i, \epsilon_i, r_i, \alpha_i)_{i \in S}$ containing the a priori mean $\eta_i \in \mathbb{R}$, the scale parameter $\epsilon_i \in \mathbb{R}^+$, the shape parameter $r_i \in \mathbb{R}^+$ and the scale parameter $\alpha_i \in \mathbb{R}^+$. The Gaussian-Inverted-Gamma distribution has been specified in a general form by Evans et al. (2000). For each state $i \in S$ the state-specific emission prior is given by a Gaussian distribution

$$N(\, \mu_i \,|\, \Theta_3 \,) := \frac{\sqrt{\epsilon_i}}{\sqrt{2\pi}\sigma_i} \exp\left(-\frac{\epsilon_i}{2} \frac{(\mu_i - \eta_i)^2}{\sigma_i^2} \right)$$

with mean η_i and standard deviation $\sigma_i / \sqrt{\epsilon_i}$ as prior distribution for the basic state-specific mean μ_i of the corresponding autoregressive mean $\mu_i^t(k)$ in combination with an Inverted-Gamma distribution

$$I_G(\, \sigma_i \,|\, \Theta_3 \,) := \frac{2{\alpha_i}^{r_i}}{\Gamma(r_i){\sigma_i}^{2r_i+1}} \exp\left(-\frac{\alpha_i}{{\sigma_i}^2} \right)$$

with shape parameter r_i and scale parameter α_i as prior of the state-specific standard deviation σ_i.

Based on that, prior knowledge can be modeled for $\mu_i^t(k)$ by choosing an a priori mean η_i and a strength ϵ_i of belief in this choice in combination with proper values for r_i and α_i to characterize the corresponding state-specific standard deviation σ_i (see emission parameter estimation formulas below and in Seifert et al. (2011) to obtain an overview of the influence of hyperparameters on the state-specific emission parameters).

1.4 Prior hyperparameters

The choice of appropriate hyperparameters for the prior distribution is generally problem-specific and depends on the size and the characteristics of the considered data set. A histogram of log-ratios (e.g. Fig. S1) can help to set the hyperparameter value of the mean η_i of each state $i \in S$ for characterizing each state of the model. For the analysis of the breast cancer and glioma gene expression data sets, the values $\eta_- := -2$, $\eta_= := 0$, and $\eta_+ := 2$ were used (each η_i is defined to be identical to the initially user-specified value of mean μ_i of state $i \in S$). These values specify that underexpressed genes are modeled by state '$-$', unchanged genes are represented by state '$=$' and that overexpressed are realized by state '$+$'. To improve the separation between these gene categories, less flexibility is allowed for the training of the emission parameters of the states '$-$' and '$+$' by using the scale parameters $\epsilon_- = \epsilon_+ := 15,000$ in comparison to $\epsilon_= := 1,000$ for the state '$=$'. To characterize each state $i \in S$ by an appropriate standard deviation, the shape parameter $r_i := 10$ and the scale parameter $\alpha_i := 10^{-4}$ were used. Finally, for each state $i \in S$ the hyperparameter $\vartheta_i := 3$ was used for the initial state prior, and for each combination of a state-context $i \in S^l$ with a next state $j \in S$ the hyperparameter $\vartheta_{ij} := 3$ was used for the transition prior. This basic initialization strategy has led to robust results in Seifert et al. (2011). That was further confirmed by an in-depth sensitivity analysis of autoregressive higher-order HMMs with respect to varying prior hyperparameter settings (see Figure 3e and f in the main manuscript). Yet, users have still the possibility to change these settings to enable the modeling of specific characteristics of other data sets. However, that was not necessary for the three different tumor data sets that we have analyzed here.

2 Appendix B: Bayesian Baum-Welch algorithm

2.1 Basics of the Bayesian Baum-Welch algorithm

The Bayesian Baum-Welch algorithm is a training procedure that iteratively determines new parameters

$$\lambda(h+1) := \underset{\lambda}{\textit{argmax}} \left(Q(\lambda \,|\, \lambda(h)) + \log(P[\lambda \,|\, \Theta]) \right)$$

for the next HMM $\lambda(h+1)$ based on the current parameters of the HMM $\lambda(h)$ ($h = 1$ initial HMM) by combining Baum's auxiliary function $Q(\lambda \,|\, \lambda(h))$ with the prior distribution $P[\lambda \,|\, \Theta]$ defined in Eqn. (1). This algorithm extends the typically used Baum-Welch training (Baum (1972); Rabiner (1989); Durbin et al. (1998)) by the integration of prior knowledge. In the following sections, Baum's auxiliary function and the prior distribution are considered to derive estimators of initial state, transition and emission parameters for the next HMM $\lambda(h+1)$. Finally, a computational scheme of the Bayesian Baum-Welch algorithm is provided.

2.2 Splitting of Baum's auxiliary function

Baum's auxiliary function provides the basis for the estimation of the model parameters using the standard Baum-Welch algorithm (Baum (1972); Rabiner (1989)). In combination with the

specified prior distribution of the HMM, Baum's auxiliary function is used to derive the basics for the parameter estimation in the context of the Bayesian Baum-Welch algorithm. Baum's auxiliary function is given by

$$Q(\,\lambda \,|\, \lambda(h)\,) := \sum_{k=1}^{K} \sum_{\vec{q} \in S^{T_k}} P[\,\vec{q} \,|\, \vec{o}(k), \lambda(h)\,] \cdot \log(P[\,\vec{o}(k), \vec{q} \,|\, \lambda\,])$$

in analogy to Seifert (2010) under consideration of the complete-data-likelihood $P[\,\vec{o}(k), \vec{q} \,|\, \lambda\,]$ of an emission sequence $\vec{o}(k) = (\vec{o}_1(k), \ldots, \vec{o}_{T_k}(k))$ and a corresponding state sequence $\vec{q} = (q_1, \ldots, q_{T_k})$. This complete-data-likelihood is given by

$$P[\,\vec{o}(k), \vec{q} \,|\, \lambda\,] := \pi_{q_1} \cdot \prod_{t=1}^{L-1} a_{\vec{q}_{1\ldots t} q_{t+1}} \cdot \prod_{t=L}^{T_k-1} a_{\vec{q}_{t-L+1\ldots t} q_{t+1}} \cdot \prod_{t=1}^{T_k} b_{q_t}(\vec{o}_t(k))$$

under an HMM $\lambda := (\vec{\pi}, A, B)$ of order L with initial state distribution $\vec{\pi}$, set of transition matrices A and emission parameters B. All these model parameters are specified in detail in the methods section of the main manuscript. Based on the complete-data-likelihood, Baum's auxiliary function can be split up into three independent functions

$$Q(\,\lambda \,|\, \lambda(h)\,) := Q_1(\,\vec{\pi} \,|\, \lambda(h)\,) + Q_2(\,A \,|\, \lambda(h)\,) + Q_3(\,B \,|\, \lambda(h)\,)$$

to represent each class of model parameters separately. Subsequently, these three functions are considered in detail and the corresponding parameter estimators or parameter estimation strategies will be derived.

2.3 Estimation of initial state parameters

To estimate the initial state probabilities for the HMM $\lambda(h+1)$, Baum's auxiliary function for the estimation of the initial state probabilities is required. This function is given by

$$\begin{aligned} Q_1(\,\vec{\pi} \,|\, \lambda(h)\,) &:= \sum_{k=1}^{K} \sum_{\vec{q} \in S^{T_k}} P[\,\vec{q} \,|\, \vec{o}(k), \lambda(h)\,] \cdot \log(\pi_{q_1}) \\ &= \sum_{i \in S} \log(\pi_i) \sum_{k=1}^{K} P[\,q_1 = i \,|\, \vec{o}(k), \lambda(h)\,] \\ &= \sum_{i \in S} \Lambda_{\pi_i} \sum_{k=1}^{K} \gamma_1^k(i) \end{aligned}$$

based on expressing the sum over all state sequences $\vec{q} \in S^{T_k}$ by two sums. The first sum considers all initial states $i \in S$, and the second sum marginalizes over all state sequences $\vec{q} \in S^{T_k}$ with initial state $q_1 = i$ leading to $P[\,q_1 = i \,|\, \vec{o}(k), \lambda(h)\,]$, which represents the state-posterior $\gamma_1^k(i)$ computed under the current HMM $\lambda(h)$ using an extended Forward-Backward algorithm for higher-order HMMs as described in Seifert (2010). Finally, the initial state probability π_i is parameterized by $\Lambda_{\pi_i} := \log(\pi_i)$ in the log-space to provide the basics for the parameter estimation.

The new initial state probability $\pi_i^{(h+1)}$ of state $i \in S$ is determined by combining Baum's auxiliary function for initial state parameters with the corresponding prior distribution $D_1(\,\vec{\pi}\,|\,\Theta_1\,)$ defined in Eqn. (2) with respect to the constraint $\sum_{i \in S} \exp(\Lambda_{\pi_i}) = 1$. This is done by applying the method of Lagrange multipliers to the auxiliary function $Q_1(\,\vec{\pi}\,|\,\lambda(h)\,) + \log(D_1(\,\vec{\pi}\,|\,\Theta_1\,)) - \delta \cdot ((\sum_{i \in S} \exp(\Lambda_{\pi_i})) - 1)$ with variable Λ_{π_i} and Lagrange multiplier δ. This leads to the new initial state probability

$$\pi_i^{(h+1)} = \frac{\left(\sum_{k=1}^{K} \gamma_1^k(i)\right) + \vartheta_i}{\left(\sum_{v \in S} \sum_{k=1}^{K} \gamma_1^k(v)\right) + \left(\sum_{v \in S} \vartheta_v\right)} \tag{5}$$

for the HMM $\lambda(h+1)$.

2.4 Estimation of transition parameters

Baum's auxiliary function for transition parameters is required to estimate the transition probabilities for the next HMM $\lambda(h+1)$. This function is specified by $Q_2(\,A\,|\,\lambda(h)\,) := \sum_{l=1}^{L} Q_2^l(\,A_l\,|\,\lambda(h)\,)$ considering each specific auxiliary function $Q_2^l(\,A_l\,|\,\lambda(h)\,)$ defined for each transition matrix $A_l \in A$ of an HMM of order L.

For a fixed state-context length $1 \leq l < L$, Baum's auxiliary function for transition matrix A_l is given by

$$\begin{aligned} Q_2^l(\,A_l\,|\,\lambda(h)\,) &:= \sum_{k=1}^{K} \sum_{\vec{q} \in S^{T_k}} P[\,\vec{q}\,|\,\vec{o}(k), \lambda(h)\,] \cdot \log\left(a_{\vec{q}_{1\ldots l} q_{l+1}}\right) \\ &= \sum_{i \in S^l} \sum_{j \in S} \log\left(a_{ij}\right) \sum_{k=1}^{K} P[\,\vec{q}_{1\ldots l} = i, q_{l+1} = j\,|\,\vec{o}(k), \lambda(h)\,] \\ &= \sum_{i \in S^l} \sum_{j \in S} \Lambda_{a_{ij}} \sum_{k=1}^{K} \varepsilon_l^k(i,j) \end{aligned}$$

substituting the sum over all state sequences $\vec{q} \in S^{T_k}$ by three sums. Two of these sums are shown explicitly and the third sum is substituted as explained subsequently. The first sum considers each current state-context $i \in S^l$. The second sum considers each next state $j \in S$. Now, a third sum is necessary to marginalize over all state sequences $\vec{q} \in S^{T_k}$ with fixed current state-context $\vec{q}_{1\ldots l} = i$ and fixed next state $q_{t+1} = j$. This third sum is simplified to its marginal distribution $\varepsilon_l^k(i,j) := P[\,\vec{q}_{1\ldots l} = i, q_{l+1} = j\,|\,\vec{o}(k), \lambda(h)\,]$ computed under the current HMM $\lambda(h)$ using an extended Forward-Backward algorithm as outlined in Seifert (2010). Finally, the transition probability a_{ij} is parameterized by $\Lambda_{a_{ij}} := log(a_{ij})$ in the log-space to provide the basics for the parameter estimation.

The new transition probability $a_{ij}^{(h+1)}$ for a transition from state-context $i \in S^l$ to state $j \in S$ is determined by combining Baum's auxiliary function for transition parameters with the corresponding prior distribution $D_2^l(\,A_l\,|\,\Theta_2^l\,)$ given in Eqn. (3) with respect to the constraint $\sum_{j \in S} \exp(\Lambda_{a_{ij}}) = 1$. This is done by applying the method of Lagrange multipliers to the auxil-

iary function $Q_2^l(\, A_l \,|\, \lambda(h)\,) + \log(D_2^l(\, A_l \,|\, \Theta_2^l\,)) - \sum_{i \in S^l} \delta_i \cdot ((\sum_{j \in S} \exp(\Lambda_{a_{ij}})) - 1)$ with variable $\Lambda_{a_{ij}}$ and Lagrange multiplier δ_i. That leads to the new transition probability

$$a_{ij}^{(h+1)} = \frac{\left(\sum_{k=1}^{K} \varepsilon_l^k(i,j) \right) + \vartheta_{ij}}{\left(\sum_{v \in S} \sum_{k=1}^{K} \varepsilon_l^k(i,v) \right) + \left(\sum_{v \in S} \vartheta_{iv} \right)} \tag{6}$$

for the HMM $\lambda(h+1)$. This transition probability is only considered for a state-transition at the fixed time-step l. The derivation of state-transition probabilities used at all time-steps $t \geq L$ considering the full memory on predecessor states is done in a similar manner and will be outlined in the following.

Baum's auxiliary function for transition matrix A_L considered for all state-transitions at time-steps $t \geq L$ is given by

$$\begin{aligned}
Q_2^L(\, A_L \,|\, \lambda(h)\,) &:= \sum_{k=1}^{K} \sum_{t=L}^{T_k-1} \sum_{\vec{q} \in S^{T_k}} P[\, \vec{q} \,|\, \vec{o}(k), \lambda(h)\,] \cdot \log\left(a_{\vec{q}_{t-L+1 \ldots t} q_{t+1}}\right) \\
&= \sum_{i \in S^L} \sum_{j \in S} \log\left(a_{ij}\right) \sum_{k=1}^{K} \sum_{t=L}^{T_k-1} P[\, \vec{q}_{t-L+1 \ldots t} = i, q_{t+1} = j \,|\, \vec{o}(k), \lambda(h)\,] \\
&= \sum_{i \in S^L} \sum_{j \in S} \Lambda_{a_{ij}} \sum_{k=1}^{K} \sum_{t=L}^{T_k-1} \varepsilon_t^k(i,j)
\end{aligned}$$

substituting the sum over all state sequences $\vec{q} \in S^{T_k}$ by three sums in analogy to the previously described derivation of the other auxiliary functions for transition parameters. The auxiliary function $Q_2^L(\, A_L \,|\, \lambda(h)\,)$ contains an additional sum over all time-steps $t \geq L$. Again, the transition probability a_{ij} is parameterized in the log-space by $\Lambda_{a_{ij}} := log(a_{ij})$.

The new transition probability $a_{ij}^{(h+1)}$ for a transition from state-context $i \in S^L$ to state $j \in S$ is determined by combining Baum's auxiliary function for transition parameters with the corresponding prior distribution $D_2^L(\, A_L \,|\, \Theta_2^L\,)$ defined in Eqn. (3) with respect to the constraint $\sum_{j \in S} \exp(\Lambda_{a_{ij}}) = 1$. This is again done by applying the method of Lagrange multipliers to the auxiliary function $Q_2^L(\, A_L \,|\, \lambda(h)\,) + \log(D_2^L(\, A_L \,|\, \Theta_2^L\,)) - \sum_{i \in S^L} \delta_i \cdot ((\sum_{j \in S} \exp(\Lambda_{a_{ij}})) - 1)$ with variable $\Lambda_{a_{ij}}$ and Lagrange multiplier δ_i. This leads to the new transition probability

$$a_{ij}^{(h+1)} = \frac{\left(\sum_{k=1}^{K} \sum_{t=L}^{T_k-1} \varepsilon_t^k(i,j) \right) + \vartheta_{ij}}{\left(\sum_{v \in S} \sum_{k=1}^{K} \sum_{t=L}^{T_k-1} \varepsilon_t^k(i,v) \right) + \left(\sum_{v \in S} \vartheta_{iv} \right)} \tag{7}$$

for the HMM $\lambda(h+1)$.

2.5 Estimation of emission parameters

In the following, Baum's auxiliary function for emission parameters is derived and prepared for the estimation of state-specific autoregressive means and standard deviations. Some basics of the parameter estimation of the autoregressive mean have already been outlined in the main manuscript. Here, detailed derivations of the parameter estimation process are given.

2.5.1 Baum's auxiliary function for emission parameters

To estimate the emission parameters for the next HMM $\lambda(h+1)$, Baum's auxiliary function for emission parameters needs to be specified. This function is given by

$$\begin{aligned} Q_3(\,B\,|\,\lambda(h)\,) &:= \sum_{k=1}^{K}\sum_{t=1}^{T_k}\sum_{\vec{q}\in S^{T_k}} P[\,\vec{q}\,|\,\vec{o}(k),\lambda(h)\,]\cdot\log\left(b_{q_t}(\vec{o}_t(k))\right) \\ &= \sum_{i\in S}\sum_{k=1}^{K}\sum_{t=1}^{T_k}\log\left(b_i(\vec{o}_t(k))\right)\cdot P[\,q_t=i\,|\,\vec{o}(k),\lambda(h)\,] \\ &= \sum_{i\in S}\sum_{k=1}^{K}\sum_{t=1}^{T_k}\log\left(b_i(\vec{o}_t(k))\right)\cdot \gamma_t^k(i) \end{aligned} \tag{8}$$

including the substitution of the sum over all state sequences $\vec{q} \in S^{T_k}$ by two sums. The first sum runs over all current states $i \in S$. Now, a second sum is required to marginalize over all state sequences $\vec{q} \in S^{T_k}$ with fixed current state $q_t = i$. The second sum can be simplified to its marginal probability $\gamma_t^k(i) := P[\,q_t = i\,|\,\vec{o}(k),\lambda(h)\,]$ representing exactly the state-posterior computed under the current HMM $\lambda(h)$ using extended Forward and Backward algorithms (Seifert (2010)).

To ease the representation of the emission parameter estimation for an autoregressive Gaussian emission distribution, some formulas and definitions given in the main manuscript are reconsidered. The expression level $\vec{o}_t(k)$ of a gene t in a profile k is modeled by a state-specific autoregressive Gaussian emission distribution of order $M \geq 0$ defined by

$$b_i(\vec{o}_t(k)) := \frac{1}{\sqrt{2\pi}\sigma_i}\exp\left(-\frac{(\vec{o}_t(k)-\mu_i^t(k))^2}{2\sigma_i^2}\right)$$

with respect to the state-specific standard deviation $\sigma_i \in \mathbb{R}^+$ and the state-specific autoregressive mean

$$\mu_i^t(k) := \mu_i + \sum_{m=1}^{M} c_{im}\cdot\vec{o}_{t-m}(k)\cdot\delta_{t,m}$$

for the expression level of gene t in profile k. Here, $\mu_i \in \mathbb{R}$ defines the basic state-specific mean and the coefficients $(c_{i1},\ldots,c_{iM}) \in \mathbb{R}^M$ are used to model the impact of predecessor expression levels on the gene-specific mean. Additionally, at the start of an emission sequence when the complete memory on M predecessor emissions does not exist, we have to truncate the modeling of dependencies by defining the function $\delta_{t,m}$ to be zero in cases where $t-m<1$ and otherwise one. The specified autoregressive Gaussian emission distribution is further used

to refine Baum's auxiliary function for emission parameters given in Eqn. (8) leading to

$$Q_3(\, B \,|\, \lambda(h)\,) = \sum_{i\in S}\sum_{k=1}^{K}\sum_{t=1}^{T_k}\left(-\log\left(\sigma_i\right) - \frac{\left(\vec{o}_t(k) - \mu_i^t(k)\right)^2}{2\sigma_i^2}\right)\cdot \gamma_t^k(i) \tag{9}$$

with respect to transformations according to logarithm rules and ignoring constant terms. This function is used subsequently in combination with the emission prior to estimate the parameters of the state-specific autoregressive mean $\mu_i^t(k)^{(h+1)}$ and the corresponding state-specific standard deviation $\sigma_i^{(h+1)}$ of the next HMM $\lambda(h+1)$.

2.5.2 Estimation of state-specific autoregressive means

The parameters of the state-specific autoregressive mean $\mu_i^t(k)$ of the autoregressive Gaussian emission density of order M of state $i \in S$ are determined by maximizing Baum's auxiliary function for emission parameters $Q_3(\, B \,|\, \lambda(h)\,)$ given in (9) in combination with the emission prior $D_3(\, B \,|\, \Theta_3\,)$ defined in (4). This is done by determining the critical point of the derivatives of $Q_3(\, B \,|\, \lambda(h)\,) + \log(D_3(\, B \,|\, \Theta_3\,))$ with respect to the basic state-specific mean μ_i and all corresponding coefficients $c_{i1}, \ldots, c_{iM}$. The resulting derivatives are set to zero and the individual terms are multiplied by σ_i^2. Additional regrouping results in a system of $M+1$ linear equations enabling to determine the parameters μ_i and $c_{i1}, \ldots, c_{iM}$ for the next model $\lambda(h+1)$. The first equation of this system of linear equations is given by

$$\begin{aligned}\epsilon_i\eta_i \;+\; \sum_{k=1}^{K}\sum_{t=1}^{T_k}\vec{o}_t(k)\cdot\gamma_t^k(i) = \;\; & \mu_i\left(\epsilon_i + \sum_{k=1}^{K}\sum_{t=1}^{T_k}\gamma_t^k(i)\right) \;+\; c_{i1}\left(\sum_{k=1}^{K}\sum_{t=2}^{T_k}\vec{o}_{t-1}(k)\cdot\gamma_t^k(i)\right) \\ & + \ldots + c_{iM}\left(\sum_{k=1}^{K}\sum_{t=M+1}^{T_k}\vec{o}_{t-M}(k)\cdot\gamma_t^k(i)\right)\end{aligned}$$

derived from the derivation of the basic state-specific mean μ_i. In addition to this, each of the corresponding M equations with fixed index $1 \leq m \leq M$ is given by

$$\begin{aligned}\sum_{k=1}^{K}\sum_{t=1}^{T_k}\vec{o}_t(k)\cdot\vec{o}_{t-m}(k)\cdot\gamma_t^k(i) = \;\; & \mu_i\left(\sum_{k=1}^{K}\sum_{t=1}^{T_k}\vec{o}_{t-m}(k)\cdot\gamma_t^k(i)\right) \\ & + c_{i1}\left(\sum_{k=1}^{K}\sum_{t=2}^{T_k}\vec{o}_{t-1}(k)\cdot\vec{o}_{t-m}(k)\cdot\gamma_t^k(i)\right) \\ & + \ldots + c_{iM}\left(\sum_{k=1}^{K}\sum_{t=M+1}^{T_k}\vec{o}_{t-M}(k)\cdot\vec{o}_{t-m}(k)\cdot\gamma_t^k(i)\right)\end{aligned}$$

derived on the basis of the derivative of $Q_3(\, B \,|\, \lambda(h)\,) + \log(D_3(\, B \,|\, \Theta_3\,))$ with respect to the corresponding coefficient c_{im}. For all equations, all components are known except the basic state-specific mean μ_i and its corresponding coefficients $c_{i1}, \ldots, c_{iM}$ for defining the autore-

gressive mean $\mu_i^t(k)^{(h+1)}$ of the next HMM $\lambda(h+1)$. These parameters can be determined by using standard solvers for systems of linear equations. Considering continuous gene expression measurements, this system of linear equations is expected to have full rank enabling to determine a unique solution for the unknown parameters μ_i and $c_{i1}, \ldots, c_{iM}$. These parameters specify the autoregressive mean $\mu_i^t(k)^{(h+1)}$ of state $i \in S$ of the next HMM $\lambda(h+1)$. We utilize the publicly available Jama package (Hicklin et al. (2012)) to compute each state-specific autoregressive mean.

2.5.3 Estimation of state-specific standard deviations

The state-specific standard deviation $\sigma_i^{(h+1)}$ of state $i \in S$ for the next HMM $\lambda(h+1)$ is determined by maximizing Baum's auxiliary function for emission parameters $Q_3(\, B \,|\, \lambda(h)\,)$ given in (9) in combination with the emission prior $D_3(\, B \,|\, \Theta_3\,)$ defined in (4). This is done by calculating the critical point of the derivative of $Q_3(\, B \,|\, \lambda(h)\,) + \log(D_3(\, B \,|\, \Theta_3\,))$ with respect to the standard deviation σ_i. This leads to the state-specific standard deviation

$$\sigma_i^{(h+1)} = \sqrt{\frac{\left(\sum_{k=1}^{K}\sum_{t=1}^{T_k} \gamma_t^k(i)\left(\vec{o}_t(k) - \mu_i^t(k)^{(h+1)}\right)^2\right) + \epsilon_i(\mu_i^{(h+1)} - \eta_i)^2 + 2\alpha_i}{\left(\sum_{k=1}^{K}\sum_{t=1}^{T_k} \gamma_t^k(i)\right) + 2r_i + 2}} \tag{10}$$

for the next HMM $\lambda(h+1)$. Here, the corresponding state-specific autoregressive mean $\mu_i^t(k)^{(h+1)}$ occurs only in the first part of the denominator, because the prior has only been specified on the corresponding basic mean $\mu_i^{(h+1)}$ to avoid time-dependencies. The obtained parameter estimator generalizes the estimator derived for the state-specific standard deviation of a non-autoregressive Gaussian emission distribution in Seifert et al. (2011).

2.6 Computational scheme of the Bayesian Baum-Welch algorithm

The computational scheme of the Bayesian Baum-Welch algorithm is specified in the following in terms of an initialization and an iteration step under consideration of the parameter estimation formulas derived in the previous sections.

- *Initialization:* Choose an initial autoregressive HMM $\lambda(1)$ with an emission process of order M and a state-transition process of order L.
- *Iteration:* For iteration steps $h = 1, 2, \ldots$
  - Use the current HMM $\lambda(h)$ to compute all state-posteriors $\gamma_t^k(i) := P[\, q_t = i \,|\, \vec{o}(k), \lambda(h)\,]$ and epsilons $\varepsilon_t^k(i,j) := P[\, \vec{q}_{\max(1,t-L+1)\ldots t} = i, q_{t+1} = j \,|\, \vec{o}(k), \lambda(h)\,]$ for each given emission sequence $\vec{o}(k)$ using the Forward-Backward algorithm adapted to higher-order HMMs in Seifert (2010).
  - Compute the optimal parameters of the next HMM $\lambda(h+1)$ with the help of these precomputations.
    1. Compute the initial state probability in (5) for each state $i \in S$.

2. Compute the transition probabilities in (6) and (7) for each state-context $i \in S^l$ with $1 \leq l \leq L$ and next state $j \in S$.
3. For each state $i \in S$, compute the basic mean $\mu_i^{(h+1)}$ and its corresponding coefficients $c_{i1}, \ldots, c_{iM}$ for the autoregressive mean $\mu_i^t(k)^{(h+1)}$ by solving the corresponding system of linear equations in section 2.5.2. Additionally, compute the standard deviation $\sigma_i^{(h+1)}$ in (10).

– Stop if the log-posterior under the next HMM $\lambda(h+1)$ has increased less than a pre-defined threshold in comparison to the log-posterior under the current HMM $\lambda(h)$, otherwise start the next iteration step with $h := h+1$.

3 Appendix C: Application of Autoregressive Higher-Order Hidden Markov Models to glioma data

Here, we consider gene expression profiles of glioblastomas by de Tayrac et al. (2009) and demonstrate that autoregressive HMMs are valuable for the identification of previously known and so far uncharacterized hotspots of differential expression. We complement this by further evaluating the identified hotspot genes in the context of a large independent cohort of glioma samples based on the Rembrandt repository by Madhavan et al. (2009).

3.1 Glioma data

A glioblastoma (glioblastoma multiforme: astrocytoma of WHO grade IV) data set by de Tayrac et al. (2009) is used to identify hotspots of over- and underexpression in glioblastomas by autoregressive HMMs. We downloaded normalized gene expression data from Gene Expression Omnibus (GEO) (GSE10878) and created average tumor-specific chromosomal gene expression profiles based on dye swap measurements. The final data set comprises 18 glioblastoma samples for which gene expression levels have been measured for 12,190 genes. The individual tumor-specific chromosomal expression profiles were structured in analogy to the breast cancer expression profiles described in the main manuscript utilizing an average normal brain reference computed based on data from de Tayrac et al. (2009). Local chromosomal dependencies between glioblastoma gene expression levels are shown in Figure 1 of the main manuscript.

Additionally, we compiled an independent glioma gene expression data set based on data from the Repository for Molecular Brain Neoplasia Data (Rembrandt, current release 1.5.9) by Madhavan et al. (2009). We performed stringent quality controls of downloaded gene expression arrays and removed all arrays with hybridization artifacts. We further did a standard Affymetrix microarray processing utilizing a customized design file from BrainArray (HGU133Plus2 version 15.0.0) in combination with GCRMA (Wu et al. (2004)) normalization. The final data set contains tumor gene expression profiles of 89 different gliomas (45 glioblastomas, 33 astrocytomas, and 11 oligodendrogliomas) for which gene expression levels of 16,282 genes are quantified in terms of log-ratios with respect to an average normal brain reference computed based on data from Rembrandt. We utilize this data set to further investigate the expression behavior of hotspots of differential expression identified in the data set by

de Tayrac et al. (2009) in the context of an independent large cohort of glioma samples. Local chromosomal dependencies between gene expression levels in gliomas are shown in Figure 1 of the main manuscript.

3.2 Identification of hotspots of differential expression in glioblastomas

Here, we apply our autoregressive HMMs to investigate their potential to identify hotspots of differential expression in glioblastomas. Therefore, we utilized publicly available glioblastoma gene expression data from de Tayrac et al. (2009). We adapted the AR(4)-HMM(2) to the tumor expression profiles of the 18 different patients based on the initial basic settings described in the methods section of the main manuscript. To reveal hotspots of differential expression across all tumors, we first determined the most likely underlying expression state of each gene in each patient by state-posterior decoding. We next ranked all genes according to their frequencies of underexpressed and overexpressed predictions across all samples and generated lists of hotspot genes. Utilizing a very stringent hotspot criterion defining that a gene must be predicted in at least 17 of 18 samples, we identified 21 hotspots of overexpression (Table S5) and 16 hotspots of underexpression (Table S6) in glioblastomas. The corresponding glioblastoma expression profiles of these genes across the different patient samples are shown in Figure S9. We note that these stringent hotpots were very similar considering different autoregressive HMMs. We performed in-depth literature studies of all hotspot genes using PubMed (www.ncbi.nlm.nih.gov/pubmed) and GeneCards (www.genecards.org) and find that seven of these genes have not been reported as hotspots of differential expression in gliomas (glioblastomas, or astrocytomas of WHO grades II or III) or other types of cancer so far. These genes are summarized in Table S7 and will be discussed together with other interesting hotspot genes in the following.

In-depth literature studies revealed that the identified hotspots of overexpression are especially enriched in genes that have previously been reported as being overexpressed in gliomas (11 of 21: glioblastomas, or astrocytomas of WHO grades II or III) or other tumors (9 of 21). So far, only one gene, CKAP2L, has not been reported as hotspot of overexpression. Currently, CKAP2L does not have an assigned annotation, but it is known to be a paralog of CKAP2, which is associated with the regulation of aneuploidy, cell cycle, and cell death in a p53-dependent manner. Thus, this gene may also be important in glioblastomas.

In contrast to these findings, literature-based studies of hotspots of underexpression revealed that a relatively large proportion of these genes has not been reported as hotspots of underexpression in gliomas or other tumors so far (6 of 16). The majority of these genes are involved in brain development, neuron function and differentiation. Three of these genes (CABP1, GABRA5, CACNG3) encode for ion channels. SNAP25 encodes for a presynaptic plasma membrane protein involved in the regulation of neurotransmitter release. MPPED1, a paralog of MPPED2, encodes for a metallophosphoesterase involved in brain development. SMPX encodes for a small protein which may play a role in the regulatory network through which muscle cells coordinate their structural and functional states during growth, adaptation and repair. Other interesting hotspots of underexpression are NEUROD6, DRD1 and SLC12A5. NEUROD6 encodes for a transcription factor involved in the development and the differentiation of the nervous system. DRD1 encodes for a G-protein coupled dopamine receptor involved

in the regulation of neuronal growth and development. SLC12A5 (alias KCC2) encodes for a membrane K-Cl cotransporter, which has been recently reported to affect cell migration, cell morphology and malignancy of cancer cell lines (Zdebik (2011)).

Interestingly, a slightly relaxed hotspot criterion of predictions in at least 15 of 18 samples has led to the identification of four hotspots of overexpression (CDCA2, CD44, OIP5, TNFRSF12A) that have recently been suggested as potential therapeutic targets in other tumors (see supporting text of Table S5 for more details). Considering the hotspots of underexpression, two other interesting genes were identified. WIF1, a known tumor suppressor inhibiting the Wnt signaling pathway, and CHD5, a potential tumor suppressor involved in chromatin remodeling and gene transcription. Additional gene ontology analysis of these extended hotspots using GOrilla (Eden et al. (2009)) revealed that hotspot genes of overexpression are significantly enriched in categories related to cell cycle, cell division, mitosis and mitotic spindle formation (p-value $<$ 1e-7). Hotspots of underexpression are significantly enriched in categories related to synapses, cell junctions and cell-cell signaling (p-value $<$ 1e-7).

3.3 Analysis of identified hotspots of differential expression in the context of different types of gliomas

Next, we analyzed the expression behavior of the identified hotspots of over- and underexpression in independent data sets taken from the Rembrandt brain tumor repository (Madhavan et al. (2009)). In addition to gene expression profiles of glioblastomas, Rembrandt also contains gene expression profiles of astrocytomas and oligodendrogliomas enabling to study how the identified hotspots behave in different types of gliomas. Therefore, we mapped the identified 21 hotspots of overexpression and the identified 16 hotspots of underexpression to the corresponding genes measured in Rembrandt. Based on that, we created a heatmap visualizing the expression behavior of the corresponding genes (Figure S10). We clearly see that the identified hotspots of over- and underexpression in glioblastomas predicted by the AR(4)-HMM(2) in data from de Tayrac et al. (2009) (Figure S9) also show very similar expression behavior in independent Rembrandt glioblastoma samples. In addition to this, some of the hotspots of overexpression are also present in astrocytomas and oligodendrogliomas, whereas others tend to be more specific for glioblastomas. Apart from that, the majority of hotspots of underexpression are also present independent of the type of glioma. Thus, based on the similarity of the expression behavior of the hotspot genes in data from de Tayrac et al. (2009) and Rembrandt, this additional evaluation clearly indicates that autoregressive HMMs are useful for the identification of differentially expressed genes in individual tumor expression profiles.

The analysis of different types of gliomas is complemented in the main manuscript by systematically investigating the overlap of top-ranking differentially expressed genes with known cancer signaling pathways.

4 Supporting Figures

4.1 Fig. S1: Histogram of log-ratios of the breast cancer gene expression data

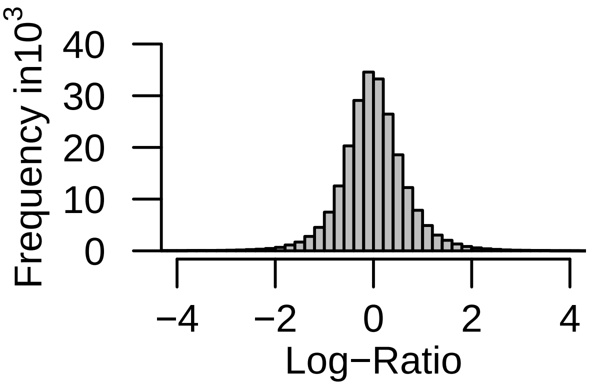

Distribution of log-ratios specifying gene expression levels in tumor compared to healthy tissue measured by Pollack et al. (2002). Log-ratios with values of about zero characterize genes with unchanged expression levels in tumor. Differentially expressed genes in tumor have log-ratios with values much less (underexpressed) or much greater (overexpressed) than zero.

4.2 Fig. S2: Three-state architecture of an AR-HMM

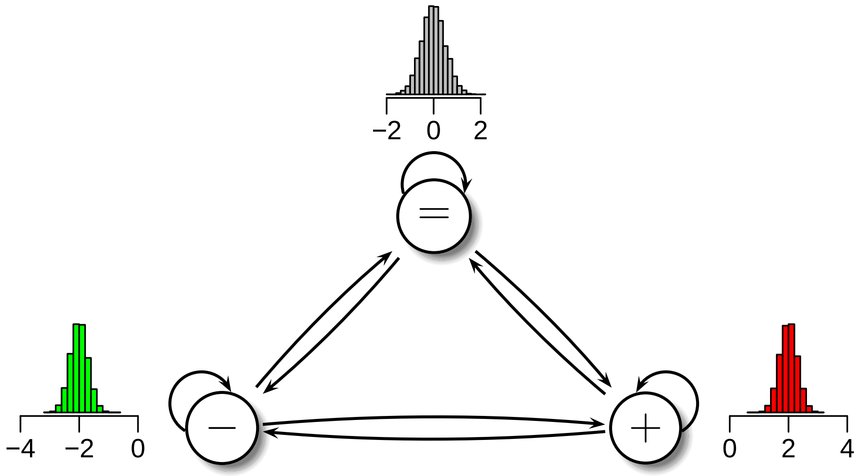

Basic three-state architecture of an autoregressive HMM for the analysis of tumor expression profiles. The states are represented by labeled circles and corresponding state-specific autoregressive Gaussian emission densities. The state '$-$' models underexpressed genes, genes with unchanged expression levels are modeled by state '$=$' and overexpressed genes are modeled by state '$+$'. Transitions between states are specified by the arrows.

4.3 Fig. S3: Impact of increased gene copy numbers on gene expression levels

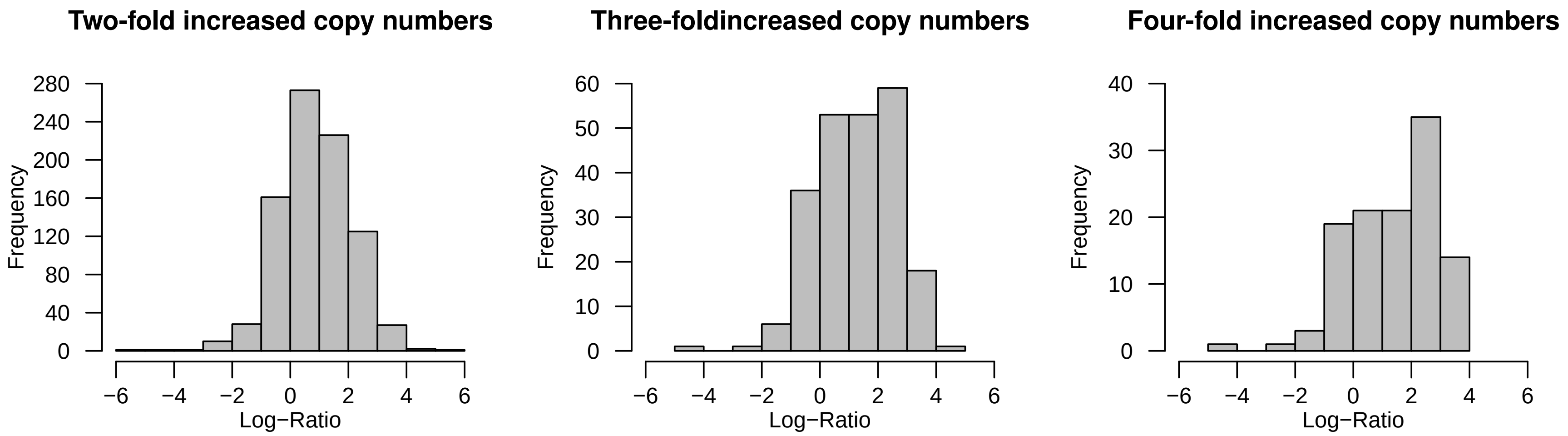

Histograms of log-ratios representing breast cancer gene expression levels of genes with at least two-, three-, or four-fold increased copy numbers in tumor compared to healthy tissue based on the breast cancer gene expression data set by Pollack et al. (2002). The majority of these genes (two-fold: 856, three-fold: 228, four-fold: 115) have increased expression levels indicated by log-ratios greater than zero. The mean log-ratio of each histogram is significantly greater than zero (t-test: p-value < 4.5e-18).

4.4 Fig. S4: Identification of overexpressed genes with known increased copy numbers by different AR-HMMs

(a) Performance on breast cancer data

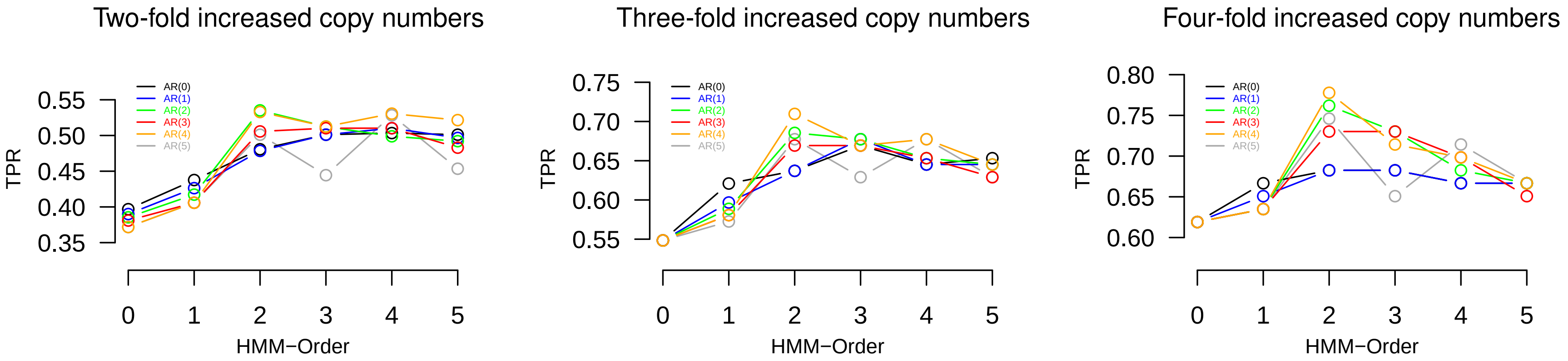

(b) Transfer to other similar breast cancer data

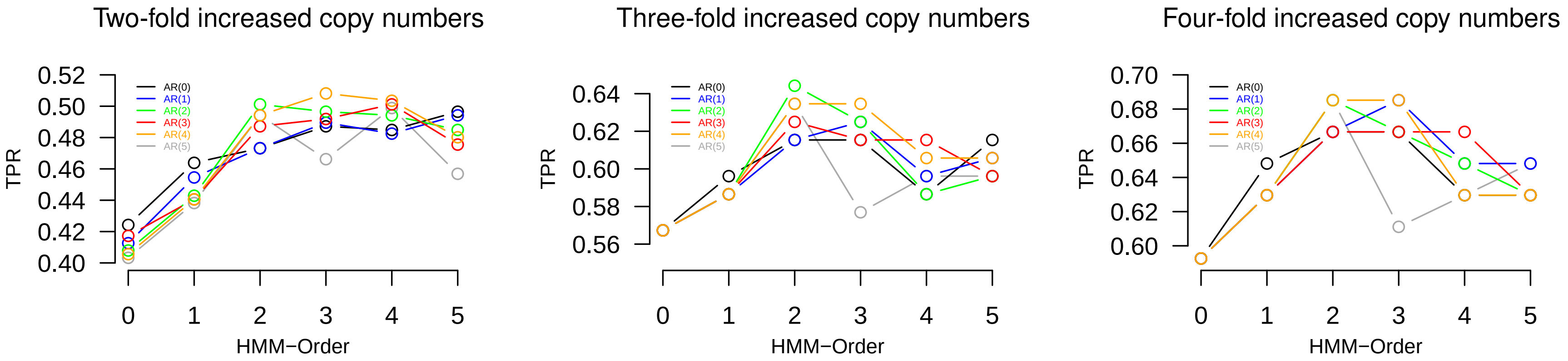

Identification of overexpressed genes with at least two-, three- or four-fold increased copy numbers by autoregressive HMMs in breast cancer gene expression data from Pollack et al. (2002). Initial HMMs with emission processes of order $M \in \{0, \ldots, 5\}$ (AR(M)) and state-transition processes of order $L \in \{0, \ldots, 5\}$ (HMM-Order) were trained by the Bayesian Baum-Welch algorithm on fifty percent of the data. The identification of candidate genes of overexpression with increased copy numbers is quantified by the true positive rate (TPR) reached at a fixed false positive rate of 5% for the training data (a) and independent test data (b).

4.5 Fig. S5: Influence of varying initial model parameter settings on the identification of overexpressed genes with known increased copy numbers by different AR-HMMs

(a) Performance on breast cancer data

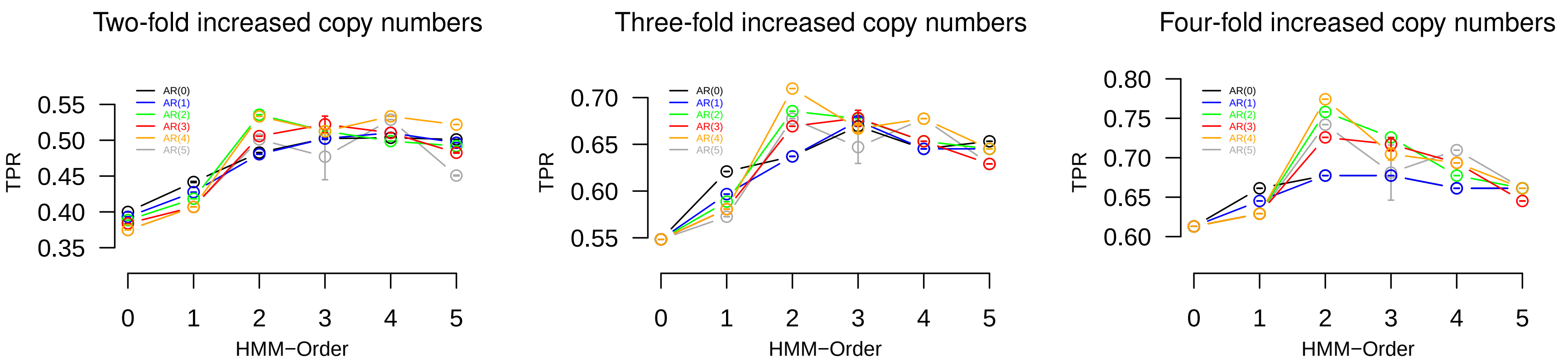

(b) Transfer to other similar breast cancer data

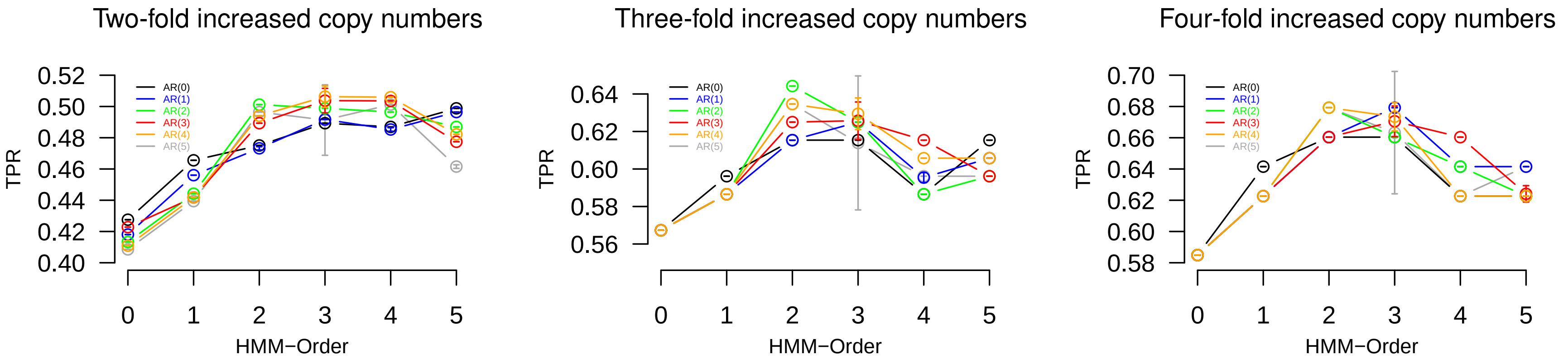

Identification of overexpressed genes with at least two-, three- or four-fold increased copy numbers by autoregressive HMMs in breast cancer gene expression data from Pollack et al. (2002). Initial HMMs were trained with respect to twelve different initial model parameter settings (Table S1) in analogy to the study in Figure S4. Average true positive rates (TPRs) and corresponding standard deviations reached at a fixed false positive rate of 5% for the different initialization scenarios are shown for the training data in (a) and for the independent test data in (b).

4.6 Fig. S6: Influence of varying prior hyperparameter settings on the identification of overexpressed genes with known increased copy numbers by different AR-HMMs

(a) Performance on breast cancer data

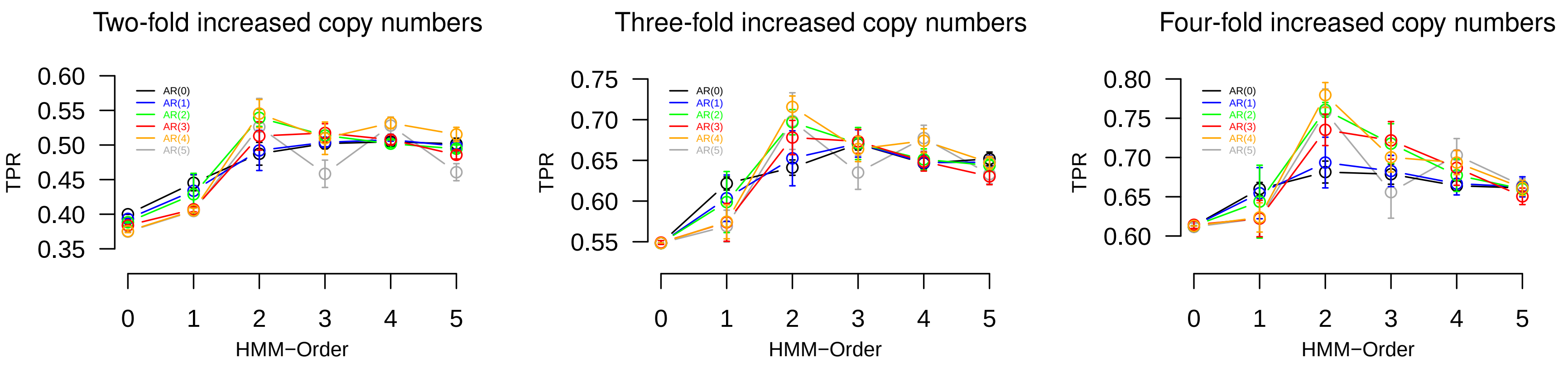

(b) Transfer to other similar breast cancer data

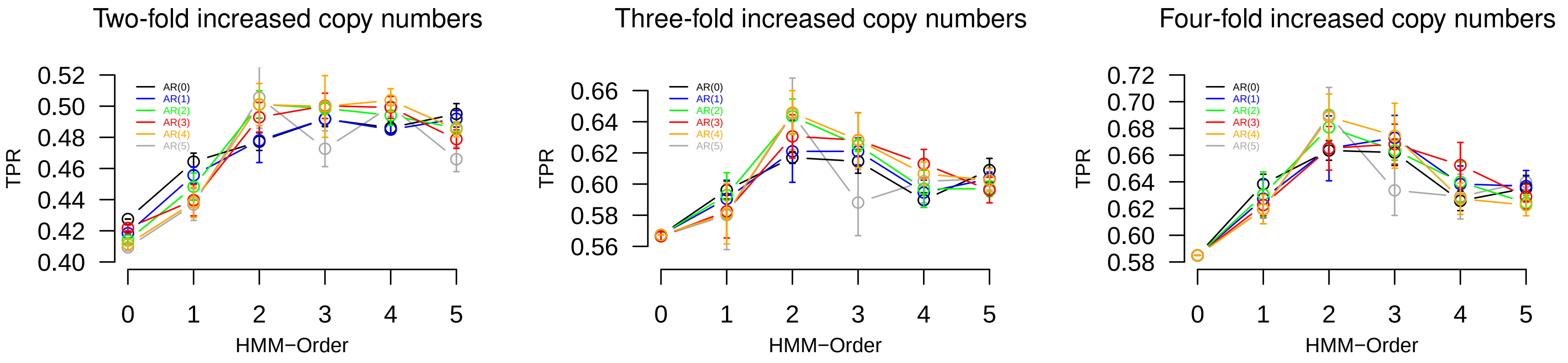

Identification of overexpressed genes with at least two-, three- or four-fold increased copy numbers by autoregressive HMMs in breast cancer gene expression data from Pollack et al. (2002). Initial HMMs were trained with respect to twelve different prior parameter settings (Table S1) in analogy to the study in Figure S4. Average true positive rates (TPRs) and corresponding standard deviations reached at a fixed false positive rate of 5% for the different initialization scenarios are shown for the training data in (a) and for the independent test data in (b).

4.7 Fig. S7: ROC curves comparing AR-HMMs to existing methods

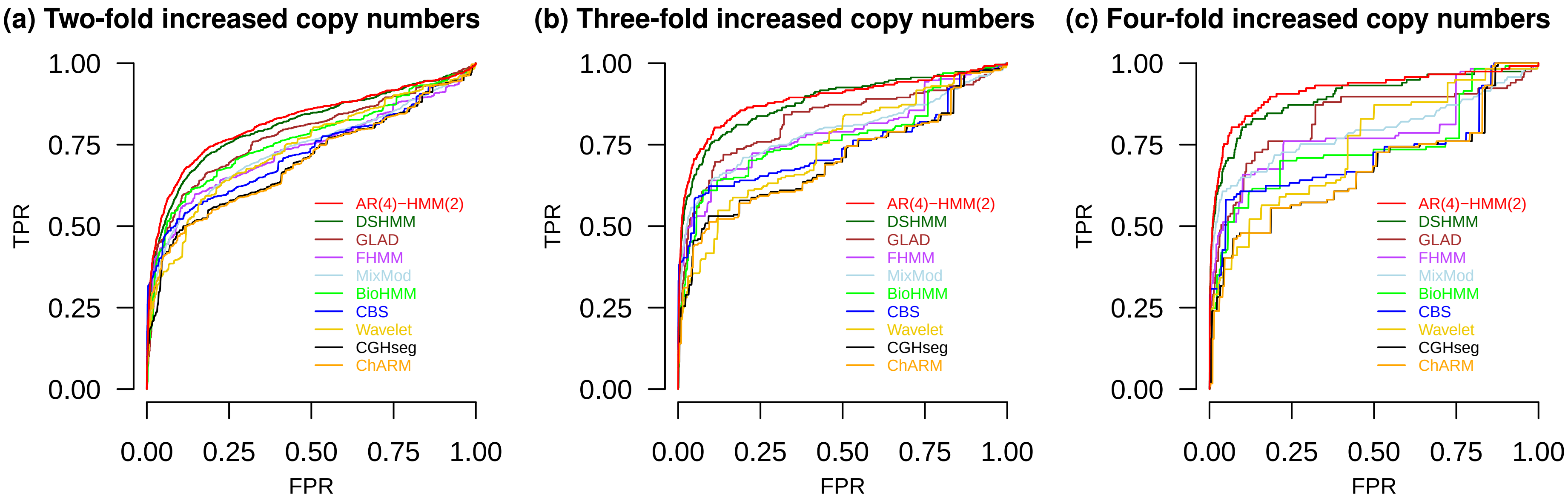

Comparison of the AR(4)-HMM(2) and related methods with respect to the identification of overexpressed genes with at least two-fold (a), three-fold (b) and four-fold (c) increased copy numbers based on breast cancer data by Pollack et al. (2002). The performance of each method is quantified by a ROC curve displaying the true positive rate (TPR) reached at different levels of false positive rates (FPR). Overall, the AR(4)-HMM(2) with a fourth-order autoregressive emission process and a second-order state-transition process reaches the best performance. Table S3 summarizes the corresponding results for fixed small FPRs and corresponding areas under the ROC curves.

4.8 Fig. S8: Pathway-based view of differentially expressed genes in the context of different Rembrandt gliomas

(a) Underexpressed genes

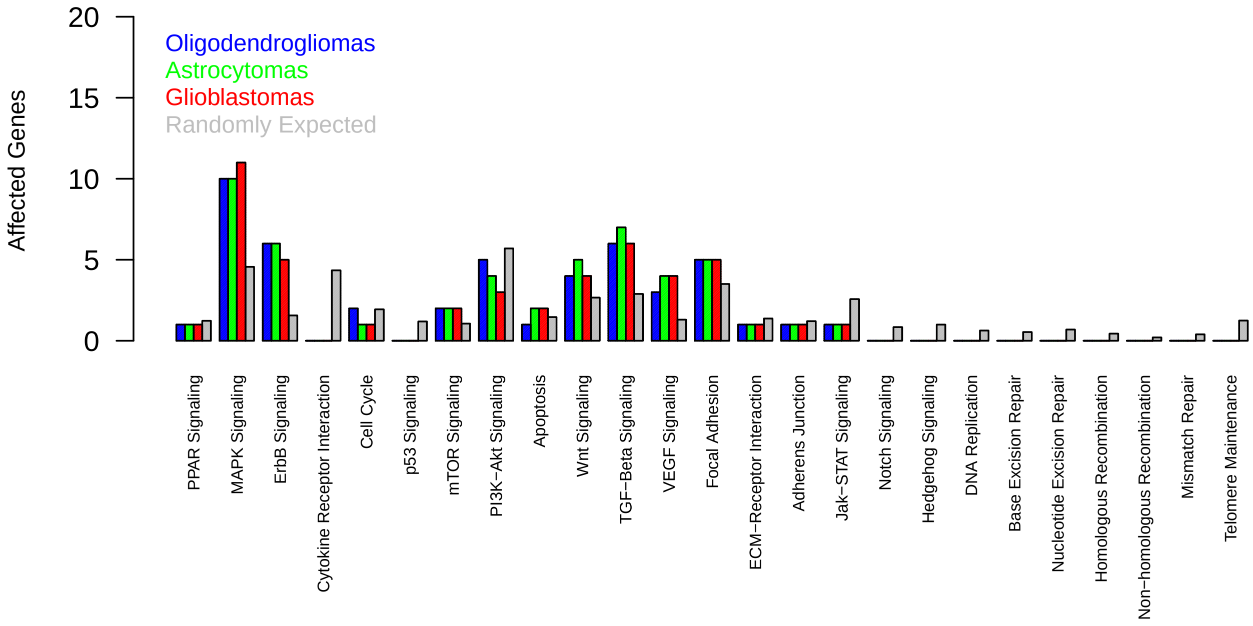

(b) Overexpressed genes

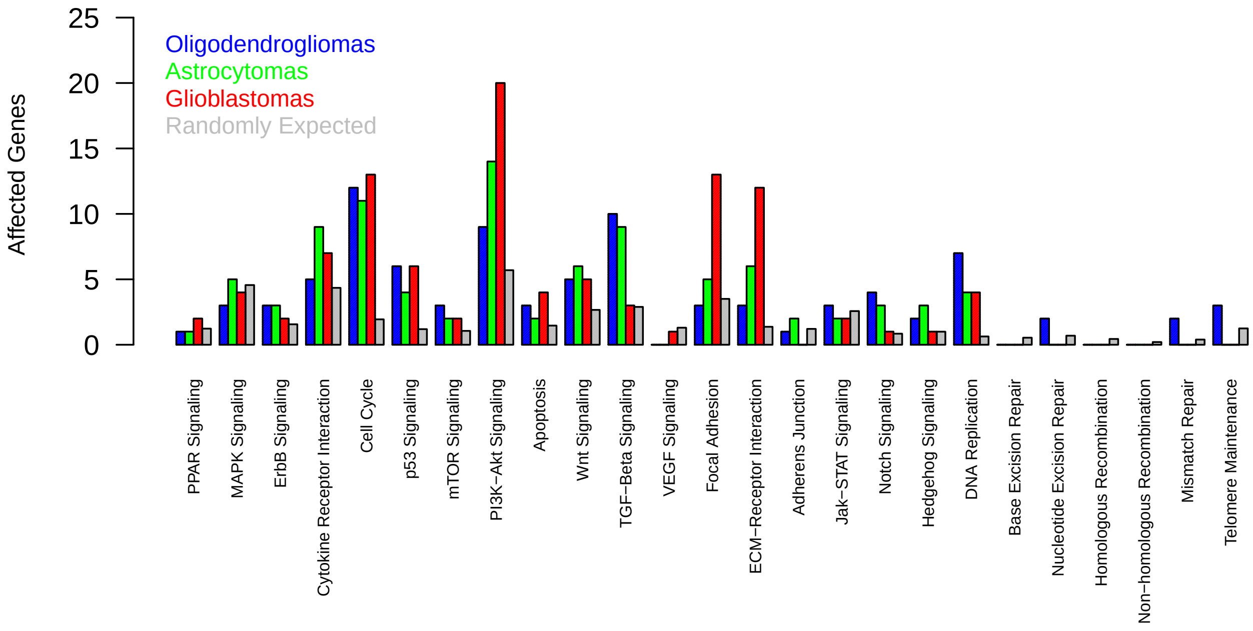

Overlap of the top 300 underexpressed genes (a) and the top 300 overexpressed genes (b) identified in the Rembrandt data set by the AR(4)-HMM(2) with known cancer signaling pathways taken from ConsensusPathDB (Kamburov et al. (2011)). Grey bars represent pathway-specific overlaps expected for randomly chosen top 300 candidate gene lists. See Figure 6 in the main manuscript for a detailed view on the most discriminative pathways between oligodendrogliomas, astrocytomas and glioblastomas. See Table S4 for an overview with functional

annotations of cancer signaling pathways.

4.9 Fig. S9: Hotspots of differential expression in glioblastomas

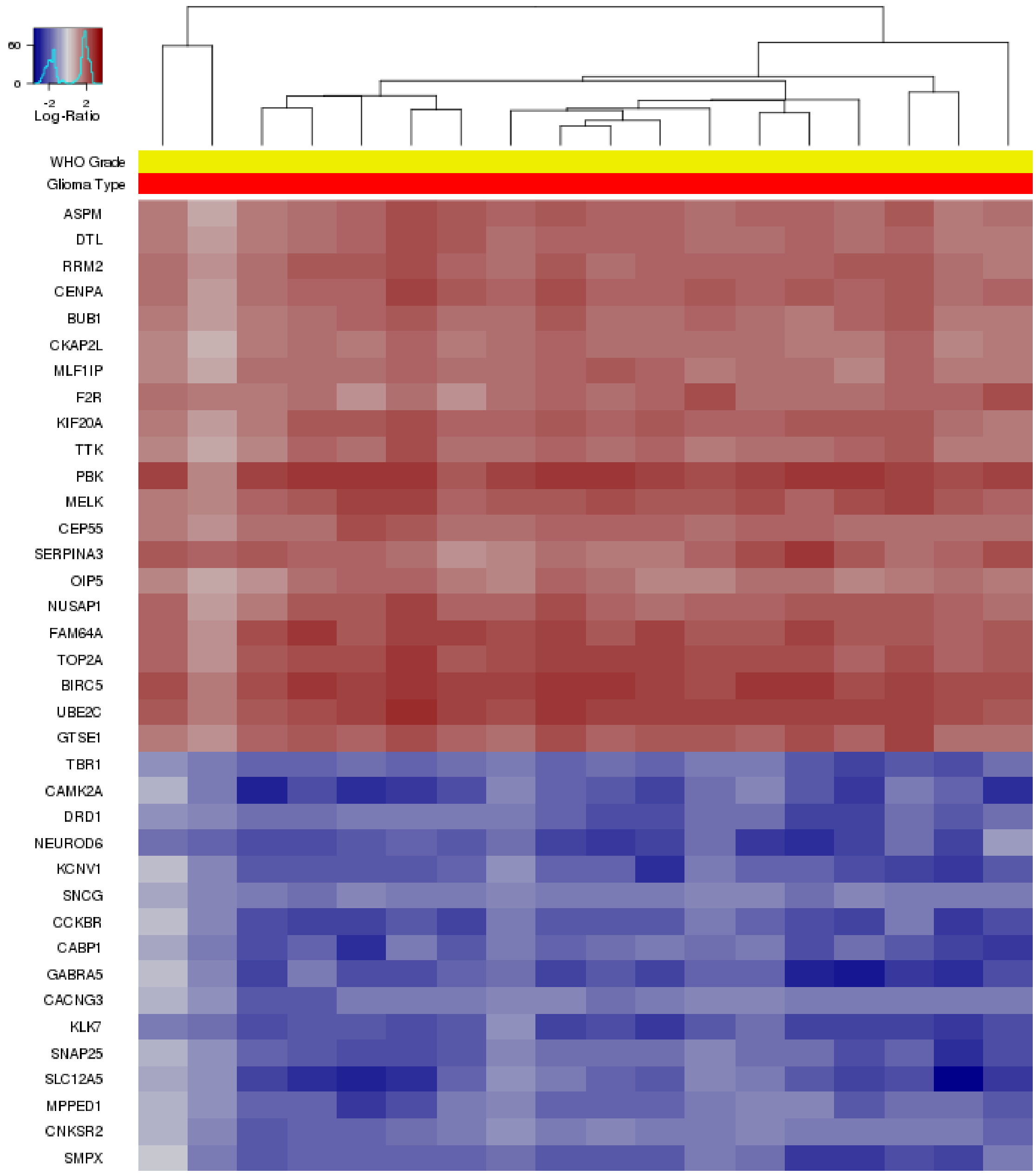

Hotspot genes of over- and underexpression identified by the AR(4)-HMM(2) in the glioblastoma data set by de Tayrac et al. (2009). The heatmap visualizes the corresponding expression profiles of the 21 hotspots of overexpression and of the 16 hotspots of underexpression that were identified in at least 17 of the 18 different tumor samples in the data set. Expression levels of individual hotspot genes are represented by log-ratios of tumor compared to normal brain. Dark red regions highlight overexpressed genes in tumor. Dark blue regions of the heatmap highlight genes underexpressed in tumor. The tumors were hierarchically clustered based on correlations of hotspot gene expression profiles in the individual tumors using average linkage clustering. More details to identified hotspot genes are given in Tables S5 and S6. See

Figure S10 for the expression profiles of the predicted hotspot genes in the context of different types of gliomas from the Rembrandt repository.

4.10 Fig. S10: Identified hotspots of differential expression in the context of different Rembrandt gliomas

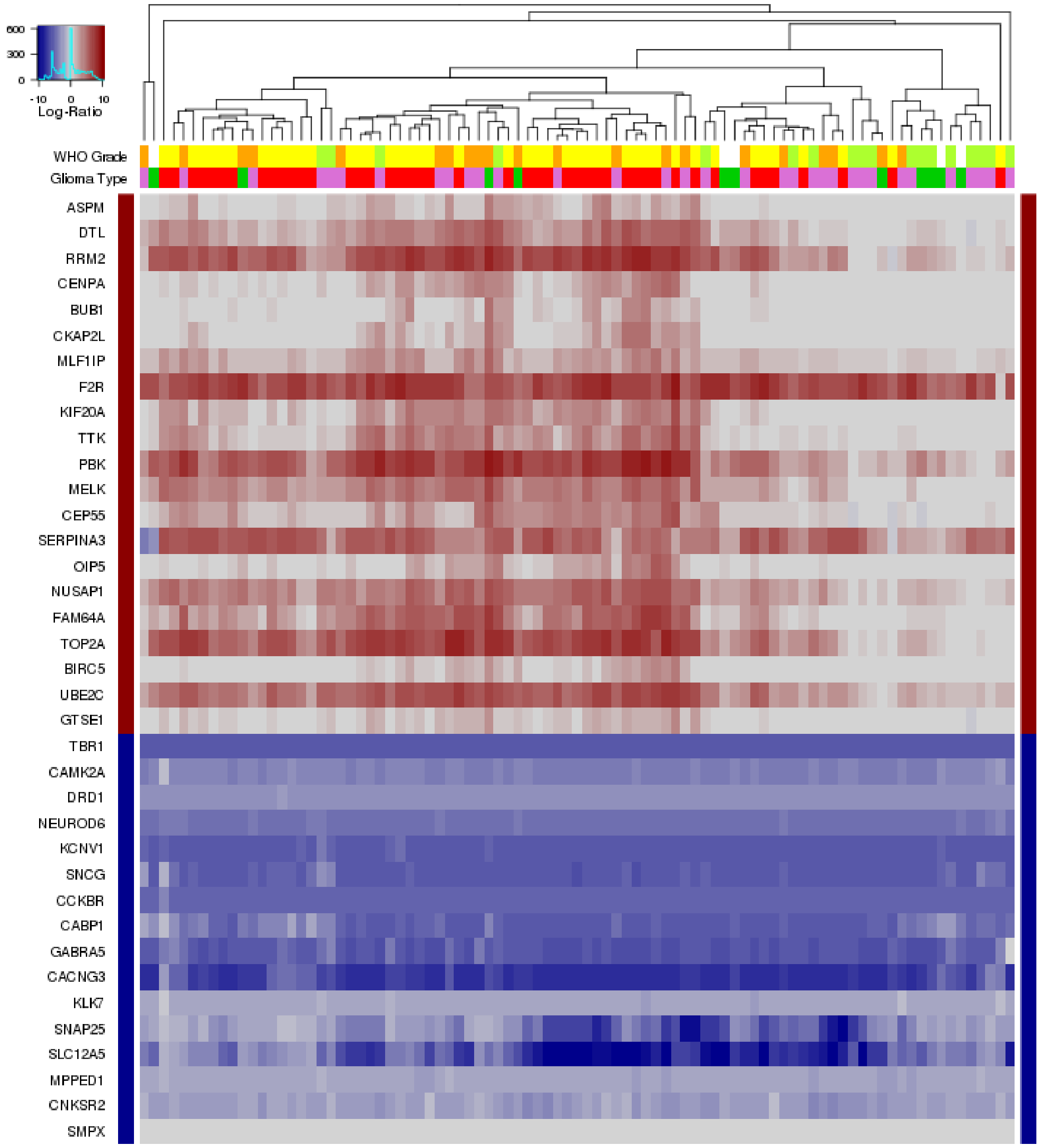

Analysis of the 21 hotspot genes of overexpression (top part of heatmap with dark red bar left and right) and the 16 hotspot genes of underexpression (bottom part of heatmap with dark blue bar left and right) identified by the AR(4)-HMM(2) in glioblastoma data from de Tayrac et al. (2009) in the context of 89 glioma samples from the Rembrandt brain tumor repository by Madhavan et al. (2009). The heatmap shows three different types of gliomas contained in Rembrandt: glioblastomas (red, 45 tumors), astrocytomas (purple, 33 tumors) and oligodendrogliomas (green, 11 tumors). The increased malignancy of these tumors is specified

according to increasing WHO grades: II (light green), III (orange), and IV (yellow). White gaps represent missing information. Expression levels of individual hotspot genes in Rembrandt tumors are represented by log-ratios of tumor compared to normal brain. Dark blue regions of the heatmap highlight genes underexpressed in tumor, grey regions show unchanged genes, and dark red regions highlight overexpressed genes in tumor. Tumors are hierarchically clustered based on the correlations of hotspot gene expression profiles in the individual tumors using average linkage clustering. The majority of the hotspots of overexpression identified in glioblastoma data from de Tayrac et al. (2009) are also strongly overexpressed in glioblastomas from Rembrandt. Some of these genes are also overexpressed in astrocytomas and oligodendrogliomas, whereas others tend to be more specifically overexpressed in glioblastomas. The hotspots of underexpression identified in the glioblastoma expression profiles from de Tayrac et al. (2009) are strongly underexpressed independent of the type of tumor in Rembrandt. More details to identified hotspot genes are given in Tables S5 and S6. See Figure S9 for the expression profiles of the predicted hotspot genes in the context of the glioblastoma data by de Tayrac et al. (2009).

5 Supporting Tables

5.1 Tab. S1: Sensitivity analysis settings

Overview of the parameter settings used to perform the sensitivity analysis to study the robustness of the predictions made by autoregressive HMMs. Table S1A summarizes the different tested initial model parameter settings. Table S1B summarizes the different tested prior hyperparameter settings. The first row always contains the basic standard settings utilized in the manuscript. Twelve different scenarios of initial model parameter settings and twelve different scenarios of prior hyperparameters were tested. In more detail, four different varying parameter settings were tested for each class of model and prior parameters for each AR(M)-HMM(L) with an autoregressive emission process of order $M \in \{0, \ldots, 5\}$ in combination with a state-transition process of order $L \in \{0, \ldots, 5\}$. We focused on systematically varying those initial model and prior parameters that are expected to have the greatest influence on the predictions of differentially expressed genes by autoregressive HMMs.

For these different scenarios, we only highlight parameters that were different from the standard settings to ease the readability. We note that the changes of the initial transition parameters in case studies 1 to 4 are directly transferred to the initial transition parameters (see Model initialization of the methods section in the main manuscript; a scale factor $s = 0.0125$ was used in case study 1 to get well-defined transition matrices, in analogy $s = 0.025$ was used in case study 2). Based on these different settings, the corresponding autoregressive HMMs were trained on fifty percent of the breast cancer gene expression data set by Pollack et al. (2002) using the Bayesian Baum-Welch algorithm. We always used the standard prior hyperparameter settings for the training of models with varying initial model parameter settings. In analogy, we always used the standard initial model parameter settings for the training of models with varying prior hyperparameters. The influences of these different scenarios on the predictions of autoregressive HMMs are summarized in Figure S5 for the different model initializations and in Figure S6 for the different prior hyperparameter settings. Generally, we find that the predictions of autoregressive HMMs are very robust.

Tab. S1A: Varying Initial Model Parameters

	Initial HMM parameters								
**Case Study**	π_-	$\pi_=$	π_+	μ_-	$\mu_=$	μ_+	σ_-	$\sigma_=$	σ_+
Standard	0.100	0.80	0.100	-2.00	0.00	2.00	0.3	0.5	0.3
1	0.025	0.95	0.025						
2	0.050	0.90	0.050						
3	0.150	0.70	0.150						
4	0.175	0.65	0.175						
5				-1.50	0.00	1.50			
6				-1.75	0.00	1.75			
7				-2.25	0.00	2.25			
8				-2.50	0.00	2.50			
9							0.1	0.5	0.1
10							0.2	0.5	0.2
11							0.4	0.5	0.4
12							0.5	0.5	0.5

Tab. S1B: Varying Prior Hyperparameters

	Prior hyperparameters								
**Case Study**	η_-	$\eta_=$	η_+	ϵ_-	$\epsilon_=$	ϵ_+	r_-	$r_=$	r_+
Standard	-2.00	0.00	2.00	15,000	1,000	15,000	10.0	10.0	10.0
13	-1.50	0.00	1.50						
14	-1.75	0.00	1.75						
15	-2.25	0.00	2.25						
16	-2.50	0.00	2.50						
17				10,000	1,000	10,000			
18				12,500	1,000	12,500			
19				17,500	1,000	17,500			
20				20,000	1,000	20,000			
21							2.5	10.0	2.5
22							5.0	10.0	5.0
23							15.0	10.0	15.0
24							20.0	10.0	20.0

5.2 Tab. S2: Summary of tested related methods

Shortcut	Method	Reference
BioHMM	Inhomogeneous first-order HMM	Marioni et al. (2006)
CBS	Circular Binary Segmentation	Olshen et al. (2004)
CGHseg	CGH segmentation	Picard et al. (2005)
ChARM	Chromosomal Aberration Region Miner	Myers et al. (2004)
DSHMM	Inhomogeneous first-order HMM	Seifert et al. (2011)
GLAD	Gain and Loss Analysis of DNA	Hupé et al. (2004)
FHMM	Homogeneous first-order HMM	Fridlyand et al. (2004)
HMM	Homogeneous first-order HMM	Seifert et al. (2011)
LFC	Log-fold change analysis	Pollack et al. (2002)
MixMod	Gaussian mixture model	Seifert et al. (2011)
SHMM	Inhomogeneous first-order HMM	Seifert et al. (2011)
Wavelet	Haar wavelet and clustering	Hsu et al. (2005)

Related methods tested for their performances of identifying overexpressed genes with known underlying gene copy number amplifications in the breast cancer gene expression data set by Pollack et al. (2002). All methods except ChARM, DSHMM, HMM, LFC, MixMod and SHMM are provided by the ADaCGH web-server (Diaz-Uriarte and Rueda (2007)). All methods except LFC and MixMod realize dependencies between neighboring genes in tumor expression profiles. BioHMM, DSHMM and SHMM additionally integrate chromosomal distances of adjacent genes on chromosomes.

5.3 Tab. S3: Global performance comparison of different methods

	**TPR at 1% FPR**			**TPR at 3% FPR**			**TPR at 5% FPR**			**TPR at 10% FPR**			**AU-ROC**		
**Shortcut**	2-fold	3-fold	4-fold	2-fold	3-fold	4-fold	2-fold	3-fold	4-fold	2-fold	3-fold	4-fold	2-fold	3-fold	4-fold
AR(4)-HMM(2)	0.32	0.49	0.51	0.46	0.63	0.67	0.54	0.71	0.75	0.64	0.77	0.82	0.82	0.89	0.92
DSHMM	0.28	0.46	0.50	0.42	0.59	0.62	0.50	0.65	0.69	0.61	0.75	0.80	0.81	0.88	0.90
SHMM	0.28	0.46	0.50	0.42	0.59	0.62	0.50	0.65	0.69	0.61	0.75	0.80	0.81	0.88	0.90
HMM	0.27	0.46	0.48	0.41	0.58	0.62	0.48	0.62	0.66	0.60	0.72	0.75	0.80	0.87	0.88
GLAD	0.14	0.26	0.28	0.42	0.46	0.44	0.46	0.54	0.50	0.56	0.64	0.61	0.78	0.82	0.83
LFC	0.20	0.36	0.43	0.34	0.52	0.56	0.41	0.56	0.61	0.52	0.63	0.65	0.74	0.79	0.79
MixMod	0.20	0.36	0.43	0.34	0.52	0.56	0.41	0.56	0.61	0.52	0.63	0.65	0.74	0.79	0.79
FHMM	0.23	0.35	0.32	0.41	0.50	0.48	0.43	0.53	0.51	0.54	0.57	0.57	0.74	0.79	0.78
Wavelet	0.20	0.21	0.25	0.31	0.31	0.32	0.35	0.36	0.37	0.40	0.42	0.44	0.74	0.75	0.75
BioHMM	0.16	0.25	0.25	0.40	0.39	0.35	0.44	0.46	0.42	0.56	0.61	0.56	0.76	0.77	0.74
CBS	0.33	0.39	0.31	0.38	0.40	0.31	0.45	0.58	0.58	0.52	0.62	0.61	0.72	0.74	0.72
CGHseg	0.19	0.25	0.24	0.24	0.29	0.28	0.40	0.46	0.40	0.49	0.53	0.48	0.70	0.71	0.68
ChARM	0.24	0.22	0.15	0.33	0.31	0.24	0.41	0.44	0.40	0.47	0.51	0.48	0.70	0.70	0.68

Identification of overexpressed genes with at least two-, three- or four-fold increased copy numbers by different methods based on all breast cancer gene expression profiles by Pollack et al. (2002). Methods are compared based on true positive rates (TPR) reached at different levels of fixed false positive rates (FPR) and based on their the area under the receiver operator characteristic curve (AU-ROC). Methods are sorted in decreasing order of corresponding AU-ROC values reached for the identification of overexpressed genes with at least four-fold increased copy numbers. We note that DSHMM and SHMM as well as MixMod and LFC reached identical performances. The AR(4)-HMM(2) reaches globally the best performance outperforming other related methods.

5.4 Tab. S4: Known cancer signaling pathways

Pathway	ID	Genes	Annotation
PPAR Signaling	path:hsa03320	67	lipid metabolism, cell proliferation and survival
MAPK Signaling	path:hsa04010	255	cell proliferation, differentiation and migration
ErbB Signaling	path:hsa04012	86	cell proliferation, differentiation, motility, survival, and migration; angiogenesis, adhesion
Cytokine Receptor Interaction	path:hsa04060	239	cell growth, differentiation, death, and communication; angiogenesis, development, immune response
Cell Cycle	path:hsa04110	111	cell division, proliferation, differentiation
p53 Signaling	path:hsa04115	66	cell cycle arrest, cellular senescence, apoptosis, central role in signaling
mTOR Signaling	path:hsa04150	59	cell growth, size, mass, differentiation, and motility
PI3K-Akt Signaling	path:hsa04151	315	cell growth, proliferation, and survival; cell cycle progression, metabolism, central role in signaling
Apoptosis	path:hsa04210	83	cell death
Wnt Signaling	path:hsa04310	145	cell polarization, proliferation, migration, and differentiation
TGF-Beta Signaling	path:hsa04350	160	cell proliferation, apoptosis, differentiation, growth, and migration; cell cycle arrest, angiogenesis
VEGF Signaling	path:hsa04370	71	angiogenesis, cell migration and survival
Focal Adhesion	path:hsa04510	194	cell motility, proliferation, differentiation, survival, and migration
ECM-Receptor Interaction	path:hsa04512	78	cell adhesion, migration, differentiation, proliferation, apoptosis, and communication
Adherens Junction	path:hsa04520	70	cell growth, differentiation, adhesion, migration, polarity and proliferation
Jak-STAT Signaling	path:hsa04630	145	cell growth, proliferation, differentiation, migration, and apoptosis
Notch Signalling	path:hsa04330	47	cell proliferation / anti-proliferation, differentiation, important in development
Hedgehog Signalling	path:hsa04340	53	cell proliferation and differentiation, important in development
DNA Replication	path:ko03030	36	duplication of DNA
Base Excision Repair	path:ko03410	31	repair of small base lesions, derived from oxidation and alkylation damages
Nucleotide Excision Repair	path:ko03420	40	repair bulky DNA damage caused by compounds, environmental carcinogens, and exposure to UV-light
Homologous Recombination	path:ko03440	26	accurate repair of DNA double-strand breaks
Non-homologous Recombination	path:ko03450	11	eliminates DNA double-strand breaks, no need for sequence homology, error-prone
Mismatch Repair	path:ko03430	22	corrects DNA mismatches from DNA replication, suppresses homol. recomb., defects: genome instability
Telomere Maintenance	reactome:M4052	71	protection of telomeres, prolonged cell survival

Known cancer signaling pathways for which we compiled corresponding pathway-specific gene list using ConsensusPathDB (Kamburov et al. (2011)). We always used KEGG-pathway entries from ConsensusPathDB, except for Telomere Maintenance for which we used the Reactome entries. The 'Gene' column represents the number of genes that could be matched to the Rembrandt microarray platform. Annotations were taken from KEGG or additional literature studies.

5.5 Tab. S5: Hotspots of overexpression in glioblastomas

Ensembl Gene ID	Chr	Start	Stop	Strand	Band	Gene	'–'	'+'
ENSG00000066279	1	197053258	197115824	-1	q31.3	ASPM	0	17
ENSG00000143476	1	212208919	212280742	1	q32.3	DTL	0	17
ENSG00000171848	2	10262455	10271545	1	p25.1	RRM2	0	17
ENSG00000115163	2	26987157	27023935	1	p23.3	CENPA	0	17
ENSG00000169679	2	111395275	111435691	-1	q13	BUB1	0	17
ENSG00000169607	2	113493930	113522254	-1	q13	CKAP2L	0	17
ENSG00000151725	4	185615772	185655287	-1	q35.1	MLF1IP	0	17
ENSG00000181104	5	76011868	76031606	1	q13.3	F2R	0	17
ENSG00000112984	5	137514408	137523404	1	q31.2	KIF20A	0	17
ENSG00000112742	6	80713604	80752244	1	q14.1	TTK	0	17
ENSG00000168078	8	27667137	27695612	-1	p21.1	PBK	0	18
ENSG00000165304	9	36572859	36677678	1	p13.2	MELK	0	17
ENSG00000138180	10	95256389	95288849	1	q23.33	CEP55	0	17
ENSG00000196136	14	95058395	95090983	1	q32.13	SERPINA3	0	17
ENSG00000104147	15	41601466	41624819	-1	q15.1	OIP5	0	16
ENSG00000137804	15	41624892	41673248	1	q15.1	NUSAP1	0	17
ENSG00000129195	17	6347735	6354789	1	p13.2	FAM64A	0	17
ENSG00000131747	17	38544768	38574408	-1	q21.2	TOP2A	0	17
ENSG00000089685	17	76210277	76221715	1	q25.3	BIRC5	0	18
ENSG00000175063	20	44441215	44445596	1	q13.12	UBE2C	0	18
ENSG00000075218	22	46692638	46726707	1	q13.31	GTSE1	0	17

This table represents hotspot genes of overexpression in the glioblastoma data set by de Tayrac et al. (2009). The table summarizes the 21 genes that have been identified as over-expressed in at least 17 of 18 tumors by state-posterior decoding of the AR(4)-HMM(2). We performed PubMed (www.ncbi.nlm.nih.gov/pubmed) literature searches and GeneCards (www.genecards.org) analysis to better characterize these genes. Dark grey rows represent genes reported as overexpressed in gliomas (glioblastomas, or astrocytomas of WHO grades II and III) in other studies (11 of 21). Light grey rows represent genes which have already been identified to be overexpressed in other tumors (9 of 21). The white row represents the gene CKAP2L that has not been reported as hotspot of overexpression in glioblastomas and which was not linked to other types of cancer (1 of 21). CKAP2L did not have an assigned annotation, but it is a paralog of CKAP2, which is associated with the regulation of aneuploidy, cell cycle, and cell death in a p53-dependent manner (http://www.genecards.org).

Additionally, we note that four genes (CDCA2, OIP5, TNFRSF12A, CD44) recently suggested as potential therapeutic target genes in other tumors (CDCA2: Uchida et al. (2013); OIP5 and TNFRSF12A: Zhou et al. (2013); CD44: Zhang et al. (2013)) have also been identified as hotspot genes of overexpression when we relaxed the hotspot criterion to at least 15 of 18 tumors. CD44 is a well-known glioma stem cell marker (Xu et al. (2010)). Gene ontology enrichment analysis of the 33 hotspot genes of overexpression in this extended list utilizing GOrilla (Eden et al. (2009)) revealed that hotspot genes of overexpression are enriched in categories related to cell cycle, cell division and mitosis (p-value < 1e-7). In accordance with that, additional analyses of this extended list showed that a large subgroup of genes (CENPA, BUB1, MLF1IP, KIF20A, CDC25C, TTK, KIF18A, OIP5, NUSAP1) is involved in mitotic spindle

formation contributing to proper chromosome segregation during mitosis.

5.6 Tab. S6: Hotspots of underexpression in glioblastomas

Ensemble Gene ID	Chr	Start	Stop	Strand	Band	Gene	'–'	'+'
ENSG00000136535	2	162272605	162282381	1	q24.2	TBR1	18	0
ENSG00000070808	5	149599054	149669854	-1	q32	CAMK2A	17	0
ENSG00000184845	5	174867675	174871163	-1	q35.2	DRD1	17	0
ENSG00000164600	7	31377075	31380508	-1	p14.3	NEUROD6	17	0
ENSG00000164794	8	110975874	110988076	-1	q23.2	KCNV1	17	0
ENSG00000173267	10	88718375	88723017	1	q23.2	SNCG	17	0
ENSG00000110148	11	6280966	6293357	1	p15.4	CCKBR	17	0
ENSG00000157782	12	121078355	121105127	1	q24.31	CABP1	17	0
ENSG00000186297	15	27111510	27194354	1	q12	GABRA5	17	0
ENSG00000006116	16	24266874	24374122	1	p12.1	CACNG3	17	0
ENSG00000169035	19	51479729	51487320	-1	q13.41	KLK7	17	0
ENSG00000132639	20	10199478	10288066	1	p12.2	SNAP25	17	0
ENSG00000124140	20	44650329	44688789	1	q13.12	SLC12A5	17	0
ENSG00000186732	22	43807202	43902800	1	q13.2	MPPED1	17	0
ENSG00000149970	X	21392536	21672813	1	p22.12	CNKSR2	17	0
ENSG00000091482	X	21724090	21776281	-1	p22.12	SMPX	17	0

This table represents hotspot genes of underexpression in the glioblastoma data set by de Tayrac et al. (2009). The table summarizes the 16 genes that have been identified as underexpressed in at least 17 of 18 tumors by state-posterior decoding of the AR(4)-HMM(2). We performed PubMed (www.ncbi.nlm.nih.gov/pubmed) literature searches and GeneCards (www.genecards.org) analysis to better characterize these genes. Dark grey rows represent genes reported as underexpressed in gliomas (glioblastomas, or astrocytomas of WHO grades II and III) or in other studies (3 of 16). Light grey rows represent genes that have already been identified to play a role in other tumors (7 of 16). White rows represent genes for which we did not find a link to gliomas or other types of cancer (6 of 16). Generally, a large proportion of identified hotspot genes is involved in brain development, neuron functions and differentiation (http://www.genecards.org). For example, four of these genes (KCNV1, CABP1, GABRA5, CACNG3) encode for ion channels. NEUROD6 encodes for a transcription factor involved in the development and the differentiation of the nervous system. DRD1 encodes for a G-protein coupled dopamine receptor involved in the regulation of neuronal growth and development. SNAP25 encodes for a presynaptic plasma membrane protein involved in the regulation of neurotransmitter release. MPPED1, a paralog of MPPED2, encodes for a metallophosphoesterase involved in brain development. SMPX encodes for a small protein which may play a role in the regulatory network through which muscle cells coordinate their structural and functional states during growth, adaptation and repair. Another interesting hotspot of underexpression is SLC12A5. SLC12A5 (alias KCC2) encodes for a membrane K-Cl cotransporter, which has been recently reported to affect cell migration, cell morphology and malignancy of cancer cell lines (Zdebik (2011)).

Additionally, we note that the tumor suppressor gene WIF1 (16 of 18) and the potential tumor suppressor gene CHD5 (15 of 18) have been identified when we relaxed the hotspot criterion.

Standard gene ontology enrichment analysis of underexpressed hotspot genes identified in at least 15 of 18 tumors (93 genes) utilizing GOrilla (Eden et al. (2009)) revealed that hotspot genes of underexpression are enriched in categories related to synapses, cell junctions and cell-cell signaling (p-value < 1e-7).

5.7 Tab. S7: Novel hotspots of underexpression in glioblastomas

Gene	Chr	Band	State	Annotation
CKAP2L	2	q13	$+$	Cytoskeleton-associated protein 2-like
CABP1	12	q24.31	$-$	Calcium-binding protein 1
GABRA5	15	q12	$-$	Gamma-aminobutyric acid receptor subunit alpha-5
CACNG3	16	p12.1	$-$	Voltage-dependent calcium channel gamma-3 subunit
SNAP25	20	p12.2	$-$	Synaptosomal-associated protein 25
MPPED1	22	q13.2	$-$	Metallophosphoesterase domain-containing protein 1
SMPX	X	p22.12	$-$	Small muscular protein

Summary of identified novel hotspots of differential expression in glioblastomas for which we did not find a link to other cancer studies based on PubMed literature searches. These hotspots were identified by the AR(4)-HMM(2) with a fourth-order autoregressive emission process and a second-order state-transition process in the glioblastoma data set by de Tayrac et al. (2009) in at least 17 out of 18 tumor samples. The table comprises one novel hotspot of overexpression $(+)$ and six novel hotspots of underexpression $(-)$. See Figures S9-10 for corresponding corresponding gene expression profiles in different glioma samples.

Authors' contributions

MS proposed autoregressive higher-order HMMs for the analysis of tumor expression profiles, implemented all algorithms, performed the studies and wrote the manuscript and the appendix. BF compiled the gene lists for the cancer signaling pathways, BF and MS added functional annotations. KA and BK contributed to the literature study of identified glioblastoma hotspot genes and helped to interpret the results obtained for the cancer signaling pathways. AD, KA and BK provided feedback to the initial draft of the manuscript and the appendix. All authors contributed to the final manuscript.

References

Baum, L. E. (1972). An inequality and associated maximization technique in statistical estimation for probabilistic functions of Markov processes. *Inequalities*, 3:1–8.

Bishop, C. M. (2006). *Pattern Recognition and Machine Learning*. In Jordan, M. and Kleinberg, J. and Schölkopf, editors, Information Science and Statistics, Springer.

de Tayrac, M. et al. (2009). Integrative Genome-Wide Analysis Reveals a Robust Genomic Glioblastoma Signature Associated with Copy Number Driving Changes in Gene Expression. *Genes, Chromosomes & Cancer*, 48:55–68.

Diaz-Uriarte, R. and Rueda, O. M. (2007). ADaCGH: A Parallelized Web-Based Application and R Package for the Analysis of aCGH Data. *PLoS ONE*, 2(8):e737.

Durbin, R. et al. (1998). *Biological sequence analysis - Probabilistic models of proteins and nucleic acids*. Cambridge University Press.

Eden, E. et al. (2009). GOrilla: A Tool For Discovery And Visualization of Enriched GO Terms in Ranked Gene Lists. *BMC Bioinformatics*, 10:48.

Evans, M., Hastings, N., and Peacock, B. (2000). *Statistical Distributions, 3rd Edition*. Wiley Series in Probability and Statistics. John Wiley & Sons, Inc.

Fridlyand, J., Snijders, A. M., Pinkel, D., Albertson, D. G., and Jain, A. N. (2004). Hidden Markov models approach to the analysis of array CGH data. *J. Multivariate Anal.*, 90:132–153.

Hicklin, J. et al. (2012). JAMA: A Java Matrix Package. http://math.nist.gov/javanumerics/jama/.

Hsu, L., Self, S. G., Grove, D., Randolph, T., Wang, K., Delrow, J. J., Loo, L., and Porter, P. (2005). Denoising array-based comparative genomic hybridization data using wavelets. *Biostatistics*, 6(2):211–226.

Hupé, P., Stransky, N., Thiery, J.-P., Radvanyi, F., and Barillot, E. (2004). Analysis of array CGH data: from signal ratio to gain and loss of DNA regions. *Bioinformatics*, 20(18):3413–3422.

Kamburov, A. et al. (2011). ConsensusPathDB: toward a more complete picture of cell biology. *Nucl. Acids Res.*, 39 (suppl 1):D712–D717.

MacKay, D. J. C. (1998). Choice of Basis for Laplace Approximation. *Machine Learning*, 33:77–86.

Madhavan, S., Zenklusen, J.-C., Kotliarov, Y., Sahmi, H., Fine, H. A., and Buetow, K. (2009). Rembrandt: Helping Personalized Medicine Become a Reality Through Integrative Translational Research. *Mol. Cancer Res.*, 7(2):157–167.

Marioni, J. C., Thorne, N. P., and Tavaré, S. (2006). BioHMM: a heterogeneous hidden Markov model for segmenting array CGH data. *Bioinformatics*, 22(9):1144–1146.

Myers, C. L. et al. (2004). Accurate detection of aneuploidies in array CGH and gene expression microarray data. *Bioinformatics*, 20(18):3533–3543.

Olshen, A. B., Venkatraman, E. S., Lucito, R., and Wigler, M. (2004). Circular binary segmentation for the analysis of array-based DNA copy number data. *Biostatistics*, 5(4):557–572.

Picard, F., Robin, S., Lavielle, M., Vaisse, C., and Daudin, J.-J. (2005). A statistical approach for array CGH data analysis. *BMC Bioinformatics*, 6(27).

Pollack, J. R. et al. (2002). Microarray analysis reveals a major direct role of DNA copy number alteration in the transcriptional program of human breast tumors. *PNAS*, 99(20):12963–12968.

Rabiner, L. R. (1989). A Tutorial on Hidden Markov Models and Selected Applications in Speech Recognition. *Proc. IEEE*, 77:257–286.

Seifert, M. (2010). Extensions of Hidden Markov Modles for the analysis of DNA microarray data. *PhD Thesis, Martin Luther University Halle,*, http://nbn-resolving.de/urn:nbn:de:gbv:3:4-4110.

Seifert, M. et al. (2011). Exploiting prior knowledge and gene distances in the analysis of tumor expression profiles by extended Hidden Markov Models. *Bioinformatics*, 27:1645–1652.

Uchida, F. et al. (2013). Overexpression of CDCA2 in Human Squamous Cell Carcinoma: Correlation with Prevention of G1 Phase Arrest and Apoptosis. *PLoS ONE*, 8(2):e56381.

Wu, Z., Irizarry, R. A., Gentleman, R., Martinez-Murillo, F., and Spencer, F. (2004). A Model-Based Background Adjustment for Oligonucleotide Expression Arrays. *J. Am. Statist. Assoc.*, 99(468):909–917.

Xu, Y. et al. (2010). CD44 attenuates activation of the hippo signaling pathway and is a prime therapeutic target for glioblastoma. *Cancer Research*, 70(6):2455–2464.

Zdebik, A. A. (2011). Beyond ion transport: KCC2 makes cells walk and talk. *J. Phisiol.*, 589:5903.

Zhang, S. et al. (2013). Targeting chronic lymphocytic leukemia cells with a humanized monoclonal antibody specific for CD44. *PNAS*, 10.1073/pnas.1221841110.

Zhou, H. et al. (2013). Antitumor Activity of a Humanized, Bivalent Immunotoxin Targeting Fn14-Positive Solid Tumors. *Cancer Research*, doi:10.1158/0008-5472.CAN-13-0187.
